# A pH-dependent bolt involving cytosine bases located in the lateral loops of antiparallel G-quadruplex structures within the SMARCA4 gene promotor

**DOI:** 10.1038/s41598-019-52311-5

**Published:** 2019-11-01

**Authors:** Sanae Benabou, Stefania Mazzini, Anna Aviñó, Ramon Eritja, Raimundo Gargallo

**Affiliations:** 1 Department of Chemical Engineering and Analytical Chemistry, University of Barcelona, Barcelona, Spain; 2 Department of Food, Environmental and Nutritional Sciences (DEFENS), University of Milan, Milan, Italy; 3 Institute for Advanced Chemistry of Catalonia (IQAC), CSIC, Networking Center on Bioengineering, Biomaterials and Nanomedicine (CIBER-BBN), Barcelona, Spain

**Keywords:** Biophysics, Structural biology, Chemistry

## Abstract

Some lung and ovarian tumors are connected to the loss of expression of SMARCA4 gene. In its promoter region, a 44-nucleotides long guanine sequence prone to form G-quadruplex structures has been studied by means of spectroscopic techniques (circular dichroism, molecular absorption and nuclear magnetic resonance), size exclusion chromatography and multivariate analysis. The results have shown that the central 21-nucleotides long sequence comprising four guanine tracts of disparate length is able to fold into a pH-dependent ensemble of G-quadruplex structures. Based on acid-base titrations and melting experiments of wild and mutated sequences, the formation of a C·C^+^ base pair between cytosine bases present at the two lateral loops is shown to promote a reduction in conformational heterogeneity, as well as an increase in thermal stability. The formation of this base pair is characterized by a pK_a_ value of 7.1 ± 0.2 at 20 °C and 150 mM KCl. This value, higher than those usually found in i-motif structures, is related to the additional stability provided by guanine tetrads in the G-quadruplex. To our knowledge, this is the first thermodynamic description of this base pair in loops of antiparallel G-quadruplex structures.

## Introduction

Nucleic acids can adopt structures other than the Watson-Crick double helix, such as the *i*-motif or G-quadruplex structures with biologically relevant roles. The i-motif is formed within cytosine-rich (C-rich) sequences, being its building block the C·C^+^ base pair. As the protonation of some cytosine bases is needed for its formation, the overall stability of the i-motif structure is strongly dependent on pH^1,2^. Because of this fact, the potential role *in vivo* of i-motif structures is still under investigation^3,4^.

**Figure 1:**
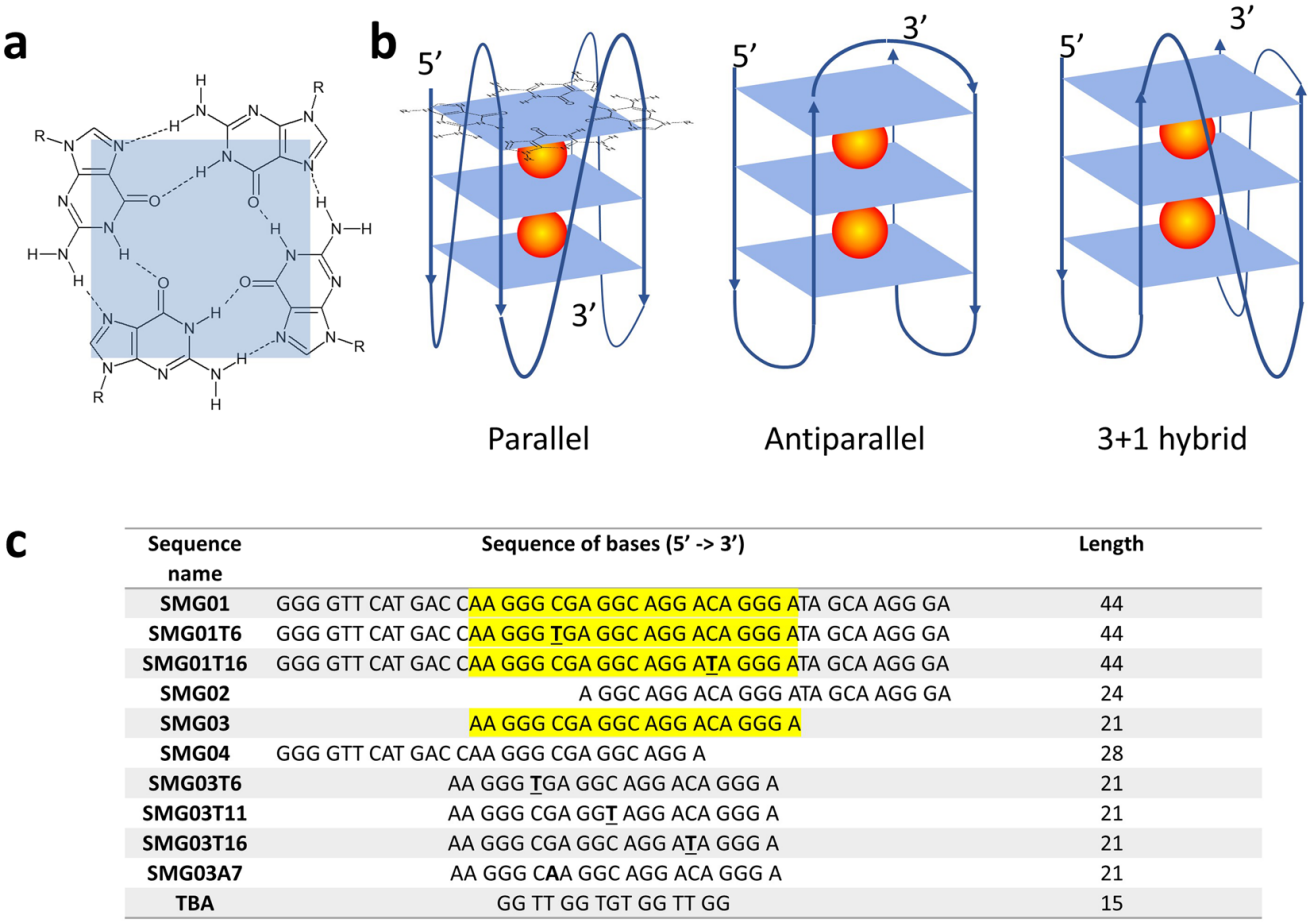
(**a**) Scheme of a G-quartet. (**b**) Arrangements of strands and loops in three typical G-quadruplex structures formed by stacking of three G-quartets. The spheres indicate cations, typically K^+^. (**c**) List of sequences studied in this work. The text highlighted in yellow indicates the position of the central SMG03 sequence into the longer SMG01 sequence. The underlined bases indicate the position of those C bases in SMG01 or SMG03 that have been mutated to T in the corresponding mutants. The suffixes ‘T6’,’T11’ and ‘T16’ indicate the position of mutated C to T bases in the central SMG03 sequence. (**a**,**b**) have been partially adapted from^56^.

On the other hand, G-quadruplexes are formed by DNA or RNA sequences that are particularly rich in guanine bases. The building block of these structures is the G-quartet (or G-tetrad), which consist on four guanine bases held together by Hoogsteen-type hydrogen bonds in a planar arrangement (Fig. 1a). G-quadruplex structures are mainly stabilized by intramolecular or intermolecular stacking of these G-quartets, as well as by electrostatic interactions with cations (such as K^+^ or Na^+^) placed within the structure. G-quadruplex structure variability depends strongly on the length of the guanine tracks, the loops connecting the stacked G-quartets, the *syn*/*anti* preference of the purine bases, and the presence of ligands or modifiers. Hence, G-quadruplexes may be classified into ‘antiparallel’, ‘parallel’ or even ‘hybrid’ arrangements (Fig. 1b)^5^. The *in vitro* formation of such structures in DNA sequences corresponding to the end of telomeres and to the promoter regions of several oncogenes has previously been shown, as well as their *in vivo* presence^6,7^. Hence, the role of G-quadruplex structures in biological processes like cancer or aging seems to be clear^8–12^. Accordingly, research is being made to identify ligands that could selectively bind to G-quadruplex structures to modulate gene expression^13,14^.

In this context, it has been identified a SMARCA4 gene involved in the development of small cell carcinoma of hypercalcemic type (SCCOHT), a rare and aggressive type of ovarian cancer^15^. The interest in the study of the regulation of this gene lies in the fact that the expression of this gene is important in controlling the cell differentiation induced by retinoic acid and glucocorticoids in both lung cancer and others. In particular, the SMARCA4 gene is related to small cell carcinoma of the ovary, hypercalcemic type (SCCOHT). The ovarian cancers are characterized by reduced SMARCA4 gene expression and/or reduced protein function and, consequently, they are sensitive to growth and/or survival inhibition by one or more compounds that restore SMARCA4 gene expression and/or protein function. In the promoter region of this gene there is a wealth of cytosine or guanine bases that could lead to the formation of folded structures, such as i-motif or G-quadruplex, respectively. In a previous work, we studied a 44-nucleotides long cytosine-rich sequence present near the promoter region (identified by us as SMC01) and proposed the formation of i-motif structures within it^16^. Here, we focused our attention on the study of the guanine-rich sequence complementary to SMC01 (SMG01, Fig. 1c). This rather long sequence includes several guanine tracts and long loops that not only could make difficult the formation of G-quadruplex structures but also would favor the formation of other structures, such as hairpins. Because of this complexity, three additional shorter sequences (SMG02-SMG04) have also been studied here.

Previously, the interaction of several G-quadruplex-forming sequences, including the central sequence SMG03, with the crystal violet ligand was studied^17^ by means of spectroscopic and separation techniques. From that study it was proposed that the polymorphism of G-quadruplex should be considered in these DNA:ligand interactions. In the present work, a detailed study on solution equilibria involving this sequence is presented. Circular dichroism (CD), nuclear magnetic resonance (NMR) and molecular absorption spectroscopies have been used with these purposes. Multivariate data analysis methods have been used to analyze spectroscopic data and to obtain qualitative and quantitative information about the role of pH or temperature in the stabilization of G-quadruplex structures. Size-exclusion chromatography (SEC) has also been used to complement the results obtained from spectroscopy.

The results show that despite the complexity of the wild sequence, folded structures, including antiparallel G-quadruplexes, may be formed within this sequence. The presence of cytosine bases at the first and third loops produces a strong stabilization of the G-quadruplex structure at near neutral pH values because of the formation of an additional C·C^+^ base pair. This stability is further enhanced in crowding media simulated by the addition of polyethylene glycol 200 (PEG200) To our knowledge, this is the first description from a thermodynamic point of view of the presence of cytosine base pairs in G-quadruplex structures.

## Results and Discussion

### Identification of the major G-quadruplex structure in SMG01

#### *In silico* prediction of structures stabilized by Watson-Crick base pairs

The formation of DNA structures stabilized by Watson-Crick base pairs at neutral pH values may be predicted by using *in silico* calculations. In this work the *mfold* method^18^ was used for this purpose. The summary of the predictions is shown in Table S1.

For SMG01, it was proposed the formation of an intramolecular structure involving five base pairs and two hairpin loops. Most of these pairs involve bases located near the 5′ end, i.e., bases that are also present in the truncated sequence SMG04, but not in SMG02 nor SMG03 (Fig. 1c). Accordingly, SMG04 could also potentially form an intramolecular structure like that depicted for SMG01. On the contrary, the hypothesized folded structures formed by SMG02 and SMG03 would be very unstable because they involve only two base pairs. At the DNA concentrations used in typical CD and molecular absorbance measurements (micromolar scale), intramolecular structures are expected to be predominant, whereas intermolecular structures, such as duplex, could be potentially formed in NMR measurements (millimolar scale).

#### *In silico* prediction of G-quadruplex structures

In a similar way to the *in silico* studied described above, the potential formation of G-quadruplex was also tested by using the Quadruplex forming G-Rich Sequences (QGRS) Mapper^19^ (Table S2). For SMG01, 44 potential G-quadruplex structures were predicted. However, the best candidate (i.e., the sequence showing the highest G-score value) was that starting in position 17, i.e., near the position 14, where the SMG03 central sequence starts. Hence, this sequence may form potentially G-quadruplex structures within the wild sequence SMG01. On the other hand, both SMG02 and SMG04 show smaller likelihood to form G-quadruplex structures.

#### Identification of G-quadruplex structures

The potential formation of G-quadruplex structures was studied by using several spectroscopies. It is known that unfolding of G-quadruplex structures monitored by molecular absorption spectroscopy is accompanied by a hypochromism at 295 nm and hyperchromism at 260 nm, whereas unfolding of hairpin or duplex structures is accompanied by hyperchromism at both wavelengths^20^. Therefore, the formation of G-quadruplex structure by a given sequence may be hypothesized from the observation of a negative band around 295 nm in the corresponding thermal difference spectrum^21^ (TDS). In this work, TDS for each sequence was calculated by subtracting the absorbance spectrum at 10 °C (where it is expected a great extension of folding) from the spectrum measured at 90 °C (where all sequences are expected to be unfolded) (Fig. S1a). From visual inspection, only the TDS spectrum of SMG03 shows the characteristics associated with the formation of G-quadruplex. The other sequences (SMG01, SMG02 and SMG04) show hyperchromism at all wavelengths, a fact that could be related with the formation of intramolecular hairpin or intermolecular duplex structures.

CD spectra of all four sequences in 20 mM sodium phosphate buffer, pH 7.1, 150 mM KCl, 10 °C are shown in Fig. S1b. In general, the CD spectrum of antiparallel structures (antiparallel G-quadruplex or i-motif) is characterized by the presence of two positive bands around 290 and 245 nm, respectively, and a negative band around 265 nm. On the other hand, the CD spectrum of parallel structures (parallel G-quadruplex, hairpin or duplex) is characterized by a positive band around 265 nm and a negative band around 245 nm, of similar intensities^22,23^. Only the CD spectrum of SMG03 may be unambiguously assigned to an antiparallel G-quadruplex structure, whereas the other spectra could correspond to parallel G-quadruplexes or intramolecular hairpins, as those predicted by *in silico* calculations.

#### Thermal stability

The stability of these folded structures against changes in temperature were studied by means of melting experiments carried out in presence of KCl. The diluted samples for measurement were prepared following the standard procedure that may be found in many studies studying G-quadruplex structures: the appropriate volumes of buffer and KCl stock solutions were added to an aliquot of the DNA stock solution, the mixture is then heated at 95 °C for 10 minutes and finally cooled overnight inside the heating block^17^.

The folded structure of SMG03 showed a clear stabilization with the addition of KCl (Table S3), in agreement with the formation of a G-quadruplex structure. The formation of a G-quadruplex implies a ΔH per G-quartet of −15 to − 25 kcal·mol^−1^ ^5,24^. In our case, the unfolding of the SMG03 sequence in 150 mM KCl required a ΔH of 23.3 kcal·mol^−1^. This value is similar to that determined for the unfolding of thrombin binding aptamer (TBA, 22.9 kcal·mol^−1^), which is a 15-nucleotides guanine-rich sequence that folds into an antiparallel, basket-type G-quadruplex structure stabilized by only two G-quartets^25,26^. Overall, these results suggest that SMG03 folds into a G-quadruplex structure involving two G-quartets, in accordance with the predicted folding by QGRS Mapper web server.

### Characterization of SMG03 folding

From the results obtained in the preliminary studies, it was clear that SMG03 sequence was able to form G-quadruplex structures in a K^+^-containing medium. Several experiments were carried out to study the dependence of the G-quadruplex stability with K^+^ concentration.

**Figure 2:**
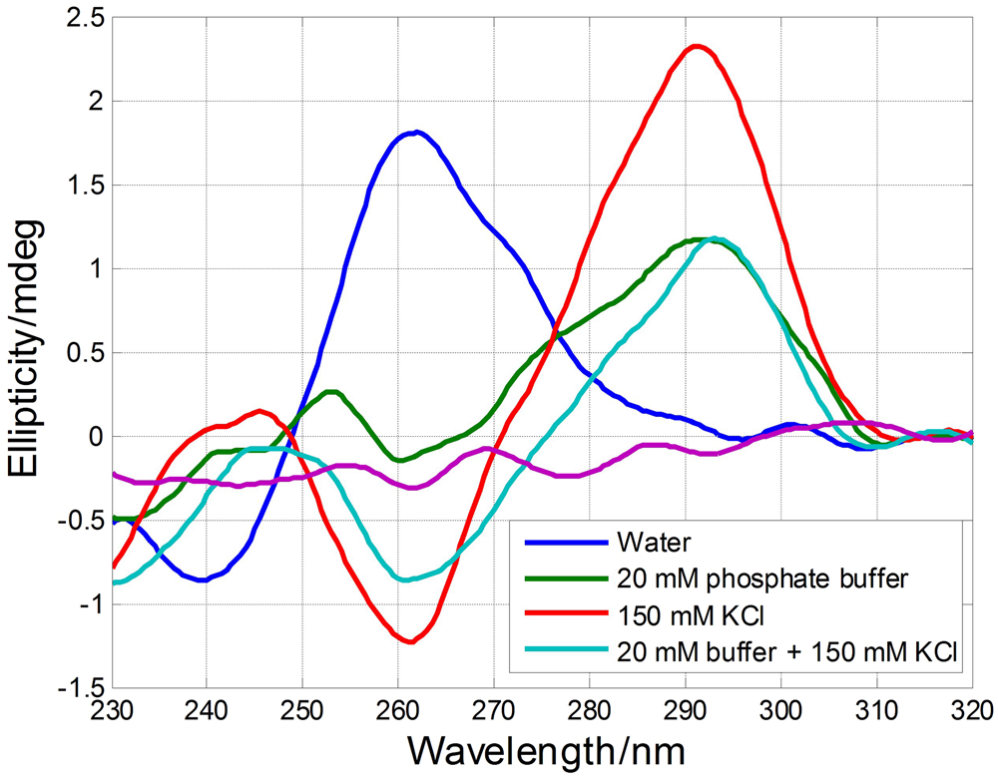
CD spectra of SMG03 measured at 10 °C in several aqueous solutions. DNA concentration was 2.0 μM.

First, CD spectra of SMG03 sequence were recorded in different aqueous solutions (Fig. 2). The spectrum measured just in water (pH 6.5, approximately) showed a positive band around 260 nm, and a weak negative band around 240 nm. These signatures could be probably related to the hairpin described by *in silico* analysis. Molecular absorption-monitored melting experiments showed a small increase of the absorbance at 260 nm, in agreement with the proposal of this hairpin structure (data not shown). Upon addition of KCl up to 150 mM (pH 6.2), the CD spectrum changed dramatically now showing positive bands at 292 and 245 nm, and a negative band at 262 nm. These signatures were very similar to those observed for guanine-rich sequences forming antiparallel G-quadruplex structures, such as TBA (Fig. 1c). When buffer was added up to 20 mM phosphate (pH 7.1, both in absence and presence of 150 mM KCl), the CD spectra showed the main characteristics of antiparallel G-quadruplex, but the overall shape was not as clear as in the absence of buffer. This fact suggested that folding of SMG03 could be pH dependent.

### Acid-base titrations

**Figure 3:**
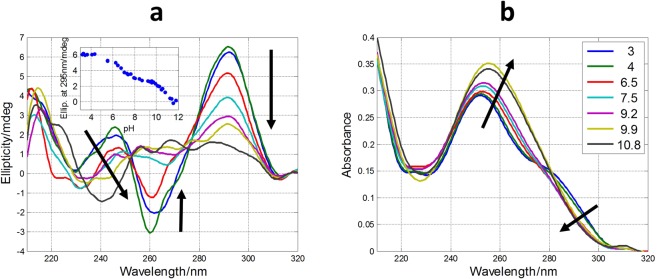
Selected CD (**a**) and molecular absorption (**b**) spectra measured along the acid-base titration of SMG03 sequence. Inset in (**a**) shows the variation of ellipticity at 292 nm with pH. Arrows indicate the sense of the main spectral changes. Inset in (**b**) indicates the pH values at which spectra were measured.

The measured CD spectra suggested the influence of pH on the folding of SMG03 into a G-quadruplex structure. To get insight on this fact, acid-base titrations monitored spectroscopically by CD and molecular absorption were carried out. The experiment consisted on the following procedure. First, a SMG03 aliquot in 150 mM KCl was placed into an optical cell and spectra and pH were measured. Stepwise additions of LiOH allowed the measurement of spectra and pH from the initial pH to pH 12, approximately. Then, successive additions of HCl from pH 12 to pH 2 allowed the measurement of the CD spectra in this pH range. No hysteresis was observed in all the studied cases, which pointed out to an intramolecular pH-induced folding. A selection of spectra measured along the titration of SMG03 sequence is shown in Fig. 3, whereas the whole set of spectra can be found in Supplementary Information (Fig. S2).

The inset in Fig. 3a shows the variation of ellipticity measured at 292 nm with pH. From this curve it is possible to deduce the existence of, at least, two acid-base transitions with pH-transition midpoints (pH_1/2_) values around 7 and 11, respectively. A third transition may be observed at pH 5. In order to obtain more information a mathematical procedure based on multivariate analysis was used to determine the number of acid-base components present along the titration in the whole pH range^27^. In addition, this methodology allows the calculation of the pH-dependent concentration profile for each one of these acid-base components, as well as the corresponding CD and molecular absorption spectra according to Eq. 4 (see “Methods”). In the case of DNA monomers (such as nitrogenous bases, nucleosides or nucleotides) an acid-base component would correspond to a chemical species that is characterized by the state of protonation of an individual acid-base group. On the other hand, in the case of DNA sequences containing a plethora of acid-base groups, such as the sequences studied here, the interpretation of an acid-base component can be more difficult as it could encompass almost concomitant changes in the state of protonation of more than one individual acid-base group. In addition, an acid-base component could also be related with a mixture of different conformations and/states of aggregation (such as monomers, dimers…) showing similar acid-base characteristics. Despite the inherent difficulties in the interpretation of these acid-base components, the fact that a very complex process, such as that shown in Fig. S2, could be explained by the contribution of very few components may provide insight to understand the pH-dependence of G-quadruplex folding.

**Figure 4:**
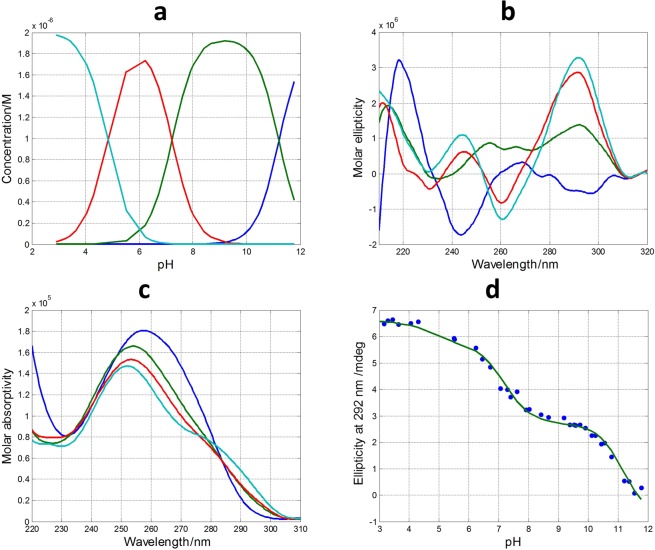
Acid-base titration of SMG03. (**a**) Calculated distribution diagram (matrix **C** in Eq. 4, see “Methods”). Calculated pure CD (**b**) and molecular absorption spectra (**c**) (matrix **S** in Eq. 4). (**d**) Experimental CD (blue symbols) and calculated with the proposed model of four acid-base components (green line) at 292 nm. Four acid-base components were considered in the calculation, the explanation of which is given in the main text. Experimental conditions were 150 mM KCl, 20 °C. DNA concentration was 2 μM.

In the case of pH-dependent folding of SMG03 sequence, four acid-base components, i.e., three acid-base transitions, were needed to explain satisfactorily the experimental data. Figure 4 shows the calculated concentration profiles for each one of the acid-base components, as well as the corresponding CD and molecular absorption spectra. Finally, Fig. 4d shows the overlap between the experimental CD data at 292 nm and the values calculated according to the proposed model of four acid-base components. Comparison of fits shown in Fig. 4d and those corresponding to a model of only three acid-base components (Fig. S2) reinforces the assumption of the existence of four components.

For the sake of comparison, the pH-dependent folding of TBA was also studied with this methodology. The analysis of the spectra recorded from pH 3 to pH 12 revealed that only two acid-base components were present in this pH range (Fig. S3 and Table 1). Obviously, the spectral features of the major component at pH values lower than 11 matched with those of an antiparallel G-quadruplex. On the other hand, the shape of the major component at pH values higher than 11 reflected the loss of the ordered structure of the G-quadruplex. Accordingly, the only transition observed was attributed to the deprotonation of 2′-deoxyguanosine and, probably, also of thymidine because both nucleosides have pK_a_ values around 9.5 ± 0.2^28^. The shift of the pK_a_ value from 9.5 to 11.1 is mainly related to the strong stabilization of guanine bases by a grid of hydrogen bonds in the G-tetrad (Fig. 1a), which hinders their deprotonation and next unfolding of the whole G-quadruplex structure.

**Table 1:** Summary of the pH-transition midpoints (pH_1/2_) and *p* values (among brackets) calculated from the acid-base titrations of DNAs.

pH_1/2_	TBA	SMG03	SMG03T6	SMG03T11	SMG03T16	SMG01	SMG01T6	SMG01T16
1^st^	11.1 ± 0.1 (2)	11.1 ± 0.1 (1)	10.7 ± 0.1 (1)	10.7 ± 0.1 (1)	11.0 ± 0.1 (1)	11.6 ± 0.1 (1)	4.2 ± 0.1 (1)	4.4 ± 0.1 (1)
2^nd^		7.1 ± 0.2 (1)	5.7 ± 0.1 (1)	6.7 ± 0.2 (1)	5.5 ± 0.2 (1)	4.7 ± 0.1 (1)		
3^rd^		4.7 ± 0.4 (1)		4.9 ± 0.4 (1)		3.8 ± 0.2 (1)		

In the case of SMG03, and similarly to that of TBA, unfolding of the G-quadruplex due to the deprotonation of 2′-deoxyguanosine nucleosides also occurs, being the pH_1/2_ of this process equal to 11.1 ± 0.1. However, the transition (*p* = 1) was not as much cooperative as in the case of TBA (*p* = 2). The shape of the major acid-base component between pH 7.1 and 11.1 (Fig. 3b, depicted in green color) shows several positive CD bands centered at 255, 265 and 290 nm, features that are clearly different from those observed in the resolved CD spectrum of the protonated species of TBA (antiparallel basket-type G-quadruplex). This fact suggests that SMG03 does not form a homogeneous antiparallel G-quadruplex structure in this pH range, but rather a potential mixture of conformations.

**Figure 5:**
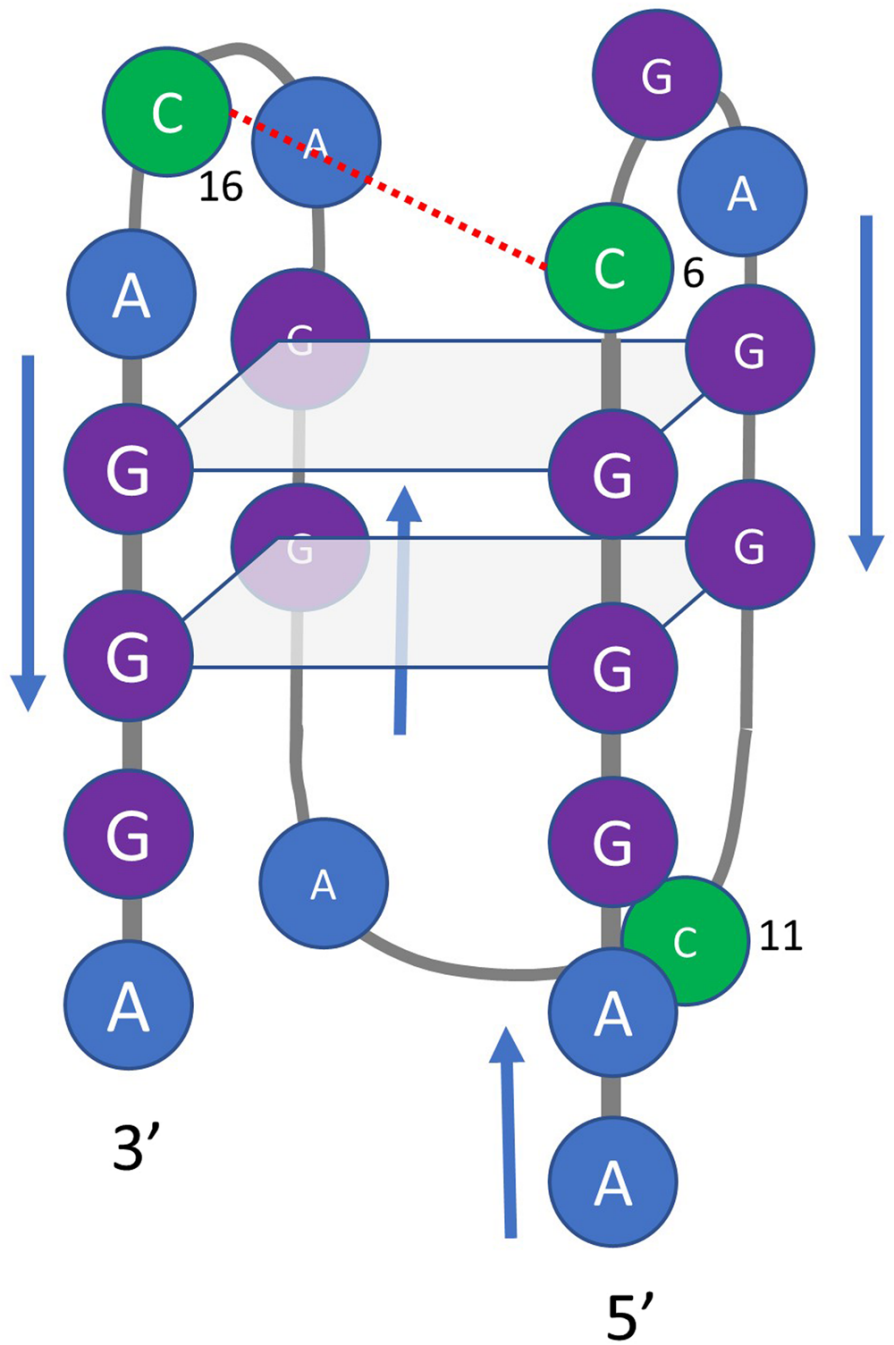
Proposed overall structure for the major acid-base component of SMG03 below pH 7.1. Guanine bases involved in the formation of the two G-tetrads are those predicted by *in silico* analysis using QGRS mapper. However, other conformers are also possible. The proposed interaction between cytosine bases is drawn with a red dotted line.

Two additional pH-dependent transitions were observed with pH_1/2_ values equal to 7.1 ± 0.2 and 4.7 ± 0.4, respectively (Fig. 4a). The latter transition was mainly related to the protonation of cytosine nucleosides, the pK_a_ of which is around 4.3^28^. The protonation of adenine residues (pK_a_ near 3.5) cannot be ruled out. On the other hand, the transition characterized by a pH_1/2_ value equal to 7.1 ± 0.2 cannot be directly related to any nucleoside as none of them has a pK_a_ value near this pH. Interestingly, this acid-base transition is accompanied by a dramatic change in CD spectra. Hence, the calculated CD spectra for the major acid-base components at pH 3 and pH 6 clearly reflect an antiparallel structure, very similar to that observed for TBA, and very different from the spectrum of the major component at pH 9. As this pH_1/2_ value (7.1) was near those observed in the study of pH-dependent folding of cytosine-rich sequences into i-motif structures^1^, we hypothesized that two of the cytosine bases in SMG03 could form a C·C^+^ base pair (Fig. 5). To test this hypothesis, three additional sequences (SMG03T6, SMG03T11 and SMG03T16) were synthesized, where three cytosine bases were mutated to thymine in order to identify the potential bases involved in that C·C^+^ base pair.

**Figure 6:**
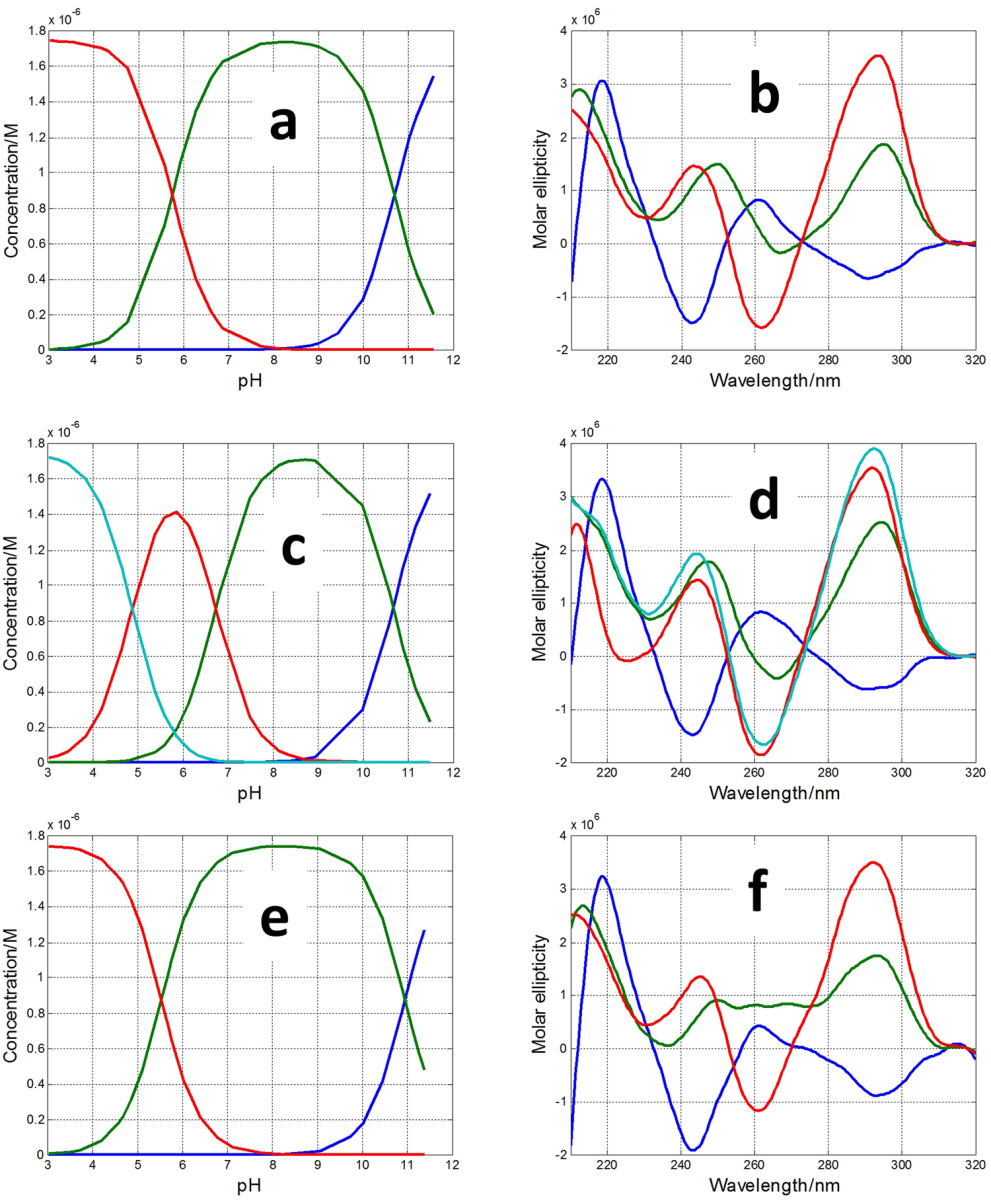
Calculated distribution diagrams (**a**,**c**,**e**) and pure spectra (**b**,**d**,**f**) for SMG03T6 (**a**,**b**), SMG03T11 (**c**,**d**) and SMG03T16 (**e**,**f**) considering three, four and three acid-base components, respectively. Experimental conditions were 150 mM KCl, 20 °C. DNA concentration was 2 μM.

Figure 6 shows the calculated distribution diagrams and pure spectra for all the acid-base components present along the pH-dependent folding of these three mutants. Whereas four acid-base components were needed to explain the set of spectra measured along the titration of SMG03T11, only three components were needed in the cases of SMG03T6 and SMG03T16 (plots of calculated *vs*. experimental ellipticity values at 292 nm are given in Figs S4–S6). Hence, the disappearance of the acid-base transition with pH_1/2_ value equal to 7.1 in the cases of SMG03T6 and SMG03T16, where the hypothesized C·C^+^ base pair cannot be formed, points to a key role of this base pair in the pH-dependent folding of SMG03 at neutral pH values.

In general, the calculated CD spectra for all acid-base components in Fig. 6 matched well with those calculated for the acid-base components present in the pH-dependent folding of the SMG03 sequence. The main differences were found on the CD spectra of the major component at pH 9 in the cases of SMG03T06 and SMG03T11, which were different from the corresponding spectra in the case of SMG03. It seems that the mutation of C6 or C11 produced a reduction of the structural diversity of the major component at pH 9 of SMG03.

### Melting studies

**Figure 7:**
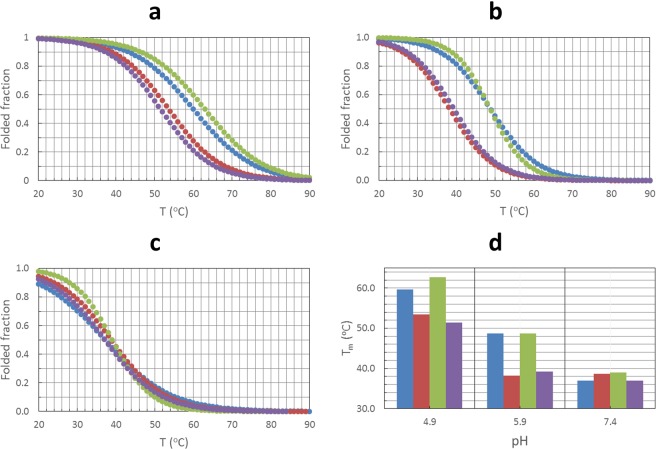
Fitted melting curves at 295 nm for SMG03, SMG03T6, SMG03T11 and SMG03T16 at pH 5.0 (**a**), 6.0 (**b**) and 7.4 (**c**). Comparison of melting temperatures (**d**). Blue: SMG03, Red: SMG03T6, Green: SMG03T11, Magenta: SMG03T16. Experimental conditions were 150 mM KCl, 20 mM phosphate or acetate buffer. DNA concentration was 2 μM.

The study of the pH-dependent folding of SMG03 suggested the existence of a C·C^+^ base pair involving C6 and C11 residues. If it existed, the formation of this base pair would have a strong influence on the thermal stability of the mutants and the wild sequence. In order to test this hypothesis, several CD- and molecular absorption-monitored melting experiments were carried out for all four sequences at several pH values (Fig. 7 and Table S4).

At pH 7.4, all sequences showed similar thermal stability, in terms of both melting temperatures (T_m_) and free Gibbs energy (ΔG_37_). At lower pH values, however, SMG03 and SMG03T11 showed clearly T_m_ values greater than those obtained for the SMG03T6 or SMG03T16 sequences. The stability of the folded structure at 37 °C is also enhanced at pH 6.0 for SMG03 and SMG03T11 in relation to other two sequences, whereas smaller differences in cooperativity are observed at pH 7.4. All these results pointed to a role of the C6 and C11 residues in the pH-dependent folding of SMG03, probably by forming a C·C^+^ base pair.

The presence of hysteresis in heating/cooling traces that could be due to slow kinetics related to the significant presence of dimeric species was checked in the case of SMG03 at pH 5.0 and pH 6.0 (Fig. S7). Both traces superimposed quite well, which ruled out the presence of significant hysteresis due to major dimeric species. Also, a melting experiment done at 10-times lower concentration (0.2 μM), which provided a T_m_ value equal to that shown in Table S4, supported this affirmation.

The proposed overall structure for the major acid-base component of SMG03 below pH 7.1 shown in Fig. 5 depicts the formation of an intramolecular C·C^+^ base pair. However, the presence of G7 could also produce a stabilization at acidic pH values due to the formation of a G·C^+^ base pair. To rule out this possibility, an additional mutated sequence SMG03A7 (AA GGG C**A**A GGC AGG ACA GGG A) was studied. The CD spectra recorded at pH 4.9 and 7.4 are very similar to those of SMG03 (Fig. S8), which reflects a similar structure. Also, CD-monitored melting experiments at pH 4.9 and 7.4 showed that this sequence unfolds at pH 4.9 with a T_m_ value (61.3 °C) similar to that determined for SMG03 (60.0 °C), which supports the hypothesis of a C·C^+^ hydrogen bonding. At neutral pH values, the determined T_m_ value for SMG03A7 (28.7 °C) is slightly lower than that of SMG03 (36.0 °C), which points out to a slight stabilization of the G-quadruplex structures at this pH due to G7, probably because of the formation of G·C base pairs.

### Size-exclusion chromatography

**Figure 8:**
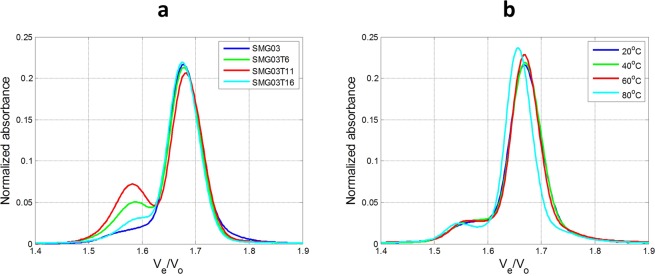
Normalized chromatograms recorded for all sequences at pH 7.1 (**a**) and normalized chromatograms of SMG03 sequence at four different temperatures (**b**). In all cases, experimental conditions were 300 mM KCl, 20 mM sodium phosphate buffer, DNA concentration was 10 μM.

Size-Exclusion Chromatography (SEC) was used to gain complementary information to that obtained from spectroscopic measurements. Figure 8 shows the normalized chromatograms at 260 nm using the relative elution volume V_e_/V_0_ as x-axis, where V_e_ is the elution volume and V_0_ is the dead volume of the used column (5.30 mL)^29,30^. This kind of normalization allows the comparison of these results to those previously published, allowing a complementary tool to assign the multimeric nature of the SEC bands. The experimental chromatograms for SMG03 at 20 °C are given in Fig. S9.

At pH 7, the chromatogram of the SMG03 sample (log_10_MW = 3.82) showed a band with elution time 11.13 minutes (V_e_/V_0_ = 1.68). According to the calibration plot built from the injection of a series of Tx standards (Fig. S9) and to previous literature^29,30^, elution of the unfolded SMG03 sequence (21 nucleotides long) should occur at 10.92 minutes. Therefore, this band at 11.13 minutes was related to a folded conformation with a smaller hydrodynamic volume than that of the unfolded SMG03. This folded conformation should be the intramolecular antiparallel structure. At pH 7.0, mutated sequences also showed this major band at 11.13 minutes and also a minor band at 10.50 minutes. According to the determined V_e_/V_0_ value (1.58) and to calibration shown in previous literature^29,30^ this band has been assigned to a dimer. The chromatogram recorded for SMG03T16 is the most similar to that of the wild sequence, whereas that of SMG03T11 showed the greater extension of this minor band.

Melting monitored by SEC indicated that dimer structures did not unfold in the experimental conditions. However, the folded monomer structure eluting at 11.13 minutes unfolded to yield a band eluting at 10.95 minutes, close to the 10.92 minutes calculated for the unfolded structure according to the calibration plot.

### NMR

**Figure 9:**
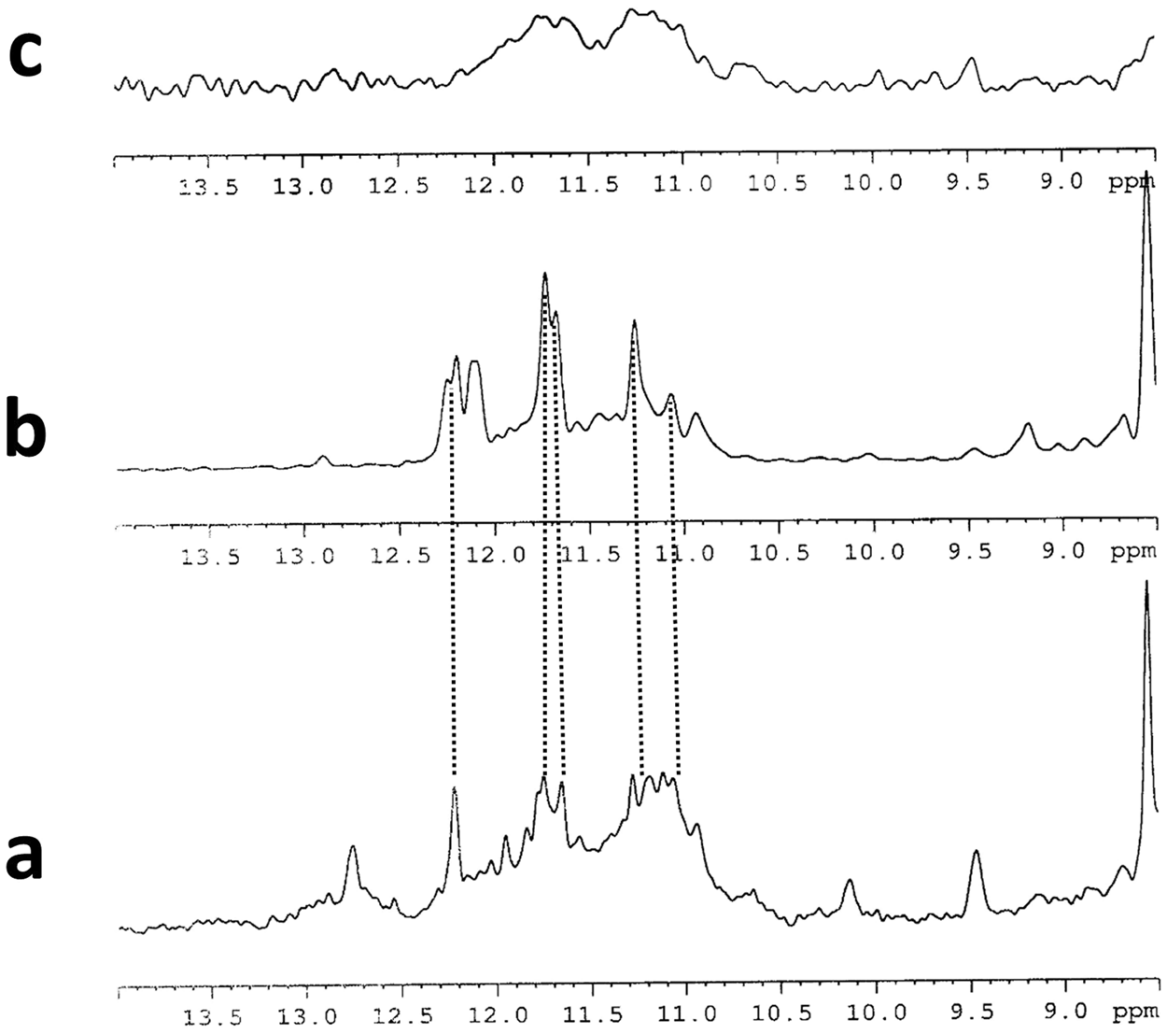
Imino proton region of ^1^H NMR spectra of (**a**) SMG03, (**b**) SMG03T11 and (**c**) SMG03T6 at 25 °C, 20 mM sodium phosphate buffer and 150 mM KCl, pH 6.0, 0.15 mM DNA concentration.

The imino proton region of the NMR spectra of the SMG03, SMG03T6 and SMG03T11 at pH 6.0 indicated the formation of quadruplex structures. Comparison of ^1^H NMR spectra of SMG03, and mutated sequences showed that G-quadruplex formation was clearly sensitive to the presence or absence of C bases at 6, 11 and 16 position (Fig. 9). In the case of SMG03T6, very broad and not defined imino protons signals between 10.2 ppm and 12 ppm revealed the coexistence of multiple G-quadruplex species in equilibrium. The broadening can derive from conformational heterogeneity due to multiple low populated conformers. In the case of SMG03 sequence, the ^1^H imino proton signals suggested the presence of better folded structures in comparison with SMG03T6. Nevertheless, an exceeding number of signals were still observed between 10.2 and 12.8 ppm. This spectrum is consistent with multiple G-quadruplex structures present in solution. The set of better defined imino proton signals in the spectrum of SMG03T11 between 10.8 ppm and 12.2 ppm indicated that a more significant amount of oligonucleotide folded into a monomeric or multimeric G-quadruplex structure. Moreover, some signals displayed the same chemical shift as those present in the spectrum of the unmodified oligonucleotide. This indicated that the major conformer of SMG03T11 adopted a structure like the one of the unmodified SMG03.

The hydrogen-bonded amino protons in the C·C^+^ base pairs were not observed in the ^1^H NMR spectra. This could be due to the intermediate-exchange processes between different conformations still present in solution at these experimental conditions.

A change in the population of different conformers of unmodified and SMG03T11 was observed when the pH value was raised from 6.0 to 9.0 (Fig. S10). The spectrum of SMG03 was characterized by broader signals in comparison with the spectrum at pH 6.0, this can be explained by the formation of multimeric quadruplex structures. This polymorphism, characterized by additional weaker peaks, was also observed in ^1^H spectrum of SMG03T11 at pH 9.0. As expected, no significant changes occur for the SMG03T6 sequence.

**Figure 10:**
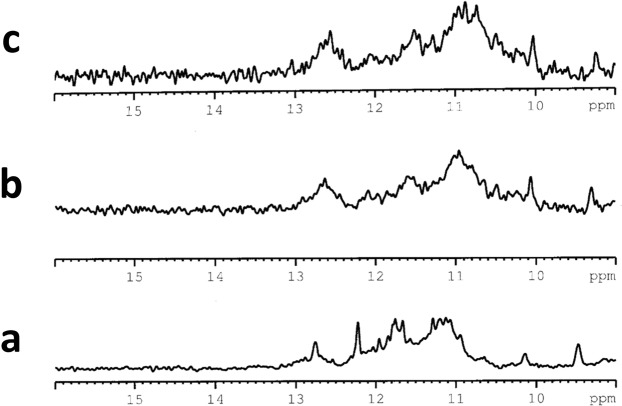
Imino proton region of ^1^H NMR spectra of SMG03 at (**a**) 25 °C, (**b**) 10 °C and (**c**) 5 °C, 20 mM sodium phosphate buffer and 150 mM KCl, pH 5.95, 0.40 mM DNA concentration.

To better study the equilibria between different G-quadruplex structures present in solution for SMG03 at 0.40 mM, the temperature was decreased from 25 °C to 5 °C (Fig. 10). Lowering the temperature, unresolved and broad signals that can be related to the formation of a higher order structures were observed.

Small variation of the ^1^H imino proton signals was observed at a concentration of 0.15 mM at 10 °C in comparison with the more concentrated sample (Fig. S11). At this temperature some sharper signals can be observed other than a hump signal, suggesting the presence of a better-defined structure together with multimeric structures.

### Stability in simulated crowding conditions

It has been reported that the cellular media are strongly crowded, and that this situation is far from being correctly simulated by *in vitro* studies in aqueous solvents^31^. To simulate the *in vivo* crowding conditions, the use of cosolutes, such as polyethylene glycol, have been suggested^32^ but also discussed^33^. In this work, the thermal stability of SMG03, its complementary cytosine-rich sequence SMC03, and of the 1:1 mixture was studied at pH 7 in a media simulated by addition of appropriate masses of PEG200 (w/v).

**Figure 11:**
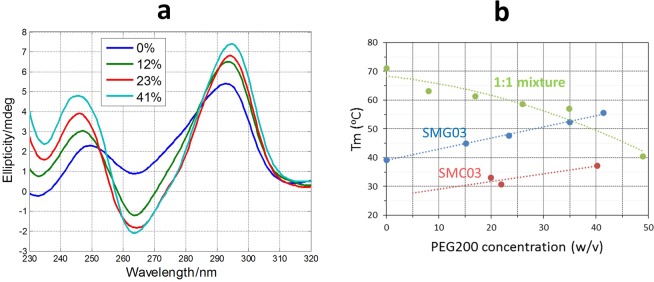
Dependence of T_m_ values for SMG03, for the complementary cytosine-rich sequence SMC03 and for the 1:1 SMG03:SMC03 mixture with PEG 200 content. Experiments were carried out in 150 mM KCl, 20 mM sodium phosphate buffer, pH 7. DNA concentration was 2 μM.

Upon increasing PEG200 concentration, the spectral features characteristics of antiparallel structures were enhanced for SMG03 sequence at pH 7.0 (Fig. 11a), which points to a stabilization of the structure in simulated crowding conditions. Concomitantly, the thermal stability of SMG03 increased linearly in the PEG200 range of concentrations tested (Fig. 11b). This stabilization of an antiparallel G-quadruplex due to the increasing concentration of PEG 200 does not agree with previous reports where it was shown that some antiparallel structures may undergo structural transitions to parallel G-quadruplex in PEG 200 containing media^34,35^. A small stabilization is also observed for the i-motif structure formed by the complementary SMC03 sequence, which hardly melts at these conditions in pure aqueous media. Overall, this tendency agrees with previous reported works where it was shown that Hoogsteen base pairs are stabilized in the presence of PEG200^32,36^. On the contrary, the thermal stability of the Watson-Crick duplex formed by the mixture of the two sequences was reduced, a fact that also agreed with previous reports^37^. At PEG 200 higher than 40% (w/v) the Watson-Crick duplex unfolds to yield a mixture of folded and unfolded SMG03 and SMC03 sequences.

### Effect of lateral nucleotides in SMG01

**Figure 12:**
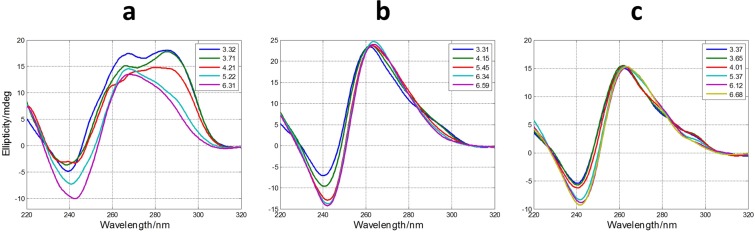
Selected CD spectra recorded along the acid-base titrations of SMG01 (**a**), SMG01T6 (**b**) and SMG01T16 (**c**). Experimental conditions were 150 mM KCl, 20 °C. DNA concentrations were 2 μM.

At this point, it has been demonstrated that the SMG03 sequence forms a major antiparallel G-quadruplex structure stabilized by the interaction of the two cytosine bases present at the lateral loops. However, the formation of this folded structure in the frame of the longer SMG01 sequence could be hindered because of the presence of additional nucleotides at both 5′ and 3′ ends. To study these effects a series of acid-base titrations of SMG01 and two mutants (SMG01T6 and SMG01T16, Fig. 1c) were carried out (Fig. 12).

The acid-base titration of SMG01 shows clearly the presence of a positive band around 290 nm that is absent in the case of the two mutants. Hence, it is concluded that the two cytosine bases that are involved in the formation of the G-quadruplex in SMG03 also had a structural role in the folding of SMG01. The multivariate analysis of all three acid-base titrations showed that the formation of the antiparallel structure within SMG01 takes place at pH values lower than 6, i.e., one pH unit below that observed for the central SMG03 sequence (Fig. S13). Therefore, besides the fact that the presence of the lateral nucleotides in SMG01 produced a destabilization of the central G-quadruplex, it did not prevent its formation.

## Discussion

As already stated, the interest in the study of the SMARCA4 gene lies in the important role in controlling cell differentiation in many cancer diseases, like small cell carcinoma of the ovary^15^. In the promoter region of this gene there is a wealth of cytosine and guanine bases that could lead to the formation of other structures than Watson-Crick duplex, such as i-motif and G-quadruplex, respectively. Hence, as these two structures have been described near the promoter regions of other oncogenes^38,39^, we decided to study the potential formation of these structures in this gene.

In a first step the solution equilibria of a cytosine-rich sequence were studied^16^. This sequence (identified by us as SMC01) is composed by 44 nucleotides and contains six tracts of cytosine bases. The length and composition of this sequence is rather unusual in the bibliography devoted to the identification of potential i-motif structures near the promoter regions of oncogenes, as it shows a few short tracts containing only two cytosine bases, together with potential long loops. On the contrary, the described i-motif structures in these regions are formed by sequences that are usually shorter and richer in cytosine bases^1,2,40^. As a result of these sequence characteristics, the proposed i-motif structures formed showed low thermal and pH stability, with T_m_ values higher than 25–30 °C only for pH values below 6.5. This low stability contrasts the reported thermal and pH stabilities of i-motif structures formed near the promoter regions of other oncogenes, such as bcl-2^41^, c-myc^42^, EGFR^43^, or Rb^44^.

In the present work, we have focused our attention on the solution equilibria of the complementary guanine-rich sequence (identified as SMG01), which is also 44 nucleotides long and contains six tracts of guanine bases. Initially, and parallel to the study of SMC01, we expected the formation of rather unstable G-quadruplex structures due mainly to the presence of long loops and short tracts of guanine bases. The initial experiments confirmed the expected trends. Hence, NMR and CD data revealed the formation by the four central tracts of guanine (SMG03) of a heterogeneous mixture of G-quadruplex structures with overall low stability in front of temperature changes. This result was rather different from the parallel, stable, and homogeneous G-quadruplex structures usually found near the promoter regions of oncogenes^45^, but similar to reported G-quadruplexes formed by short guanine tracts^46^.

On the other hand, CD spectra recorded in different media suggested a key role of pH on the folding of G-quadruplex structures in SMG03 sequence, a variable that is not usually considered in the study of these structures. Then, we planned a series of spectroscopically monitored acid-base titrations of SMG03 to gain quantitative and spectral information about the influence of pH on the formation of G-quadruplex structures. Surprisingly, the obtained results showed the presence of a conformational transition associated to an acid-base transition with a pK_a_ value 7.1 ± 0.2 at 20 °C and 150 mM KCl. According to CD data, at pH lower than this pK_a_ the homogeneity of the G-quadruplex population is clearly enhanced, producing antiparallel structures, whereas at higher pH values there is a clear loss of homogeneity. After ruling out several possibilities to explain this fact, we focused our attention on two cytosine bases potentially present at the lateral loops of the antiparallel structure. We hypothesized that these cytosine bases could form a C·C^+^ base pair that could lock the antiparallel structure. Further studies done with mutants not showing these cytosine bases confirmed the importance of this C·C^+^ base pair at neutral pH to produce an antiparallel and rather homogeneous structure with higher thermal stability.

The pK_a_ value of free cytosine is around 4.5 at 25 °C^2^. Accordingly, the formation of C·C^+^ base pairs by monomers could only be possible at pH values lower than 5.5, approximately. However, i-motif structures stabilized by these base pairs have been described at neutral pH values, both *in vitro* and *in vivo*^3,47^. However, to our knowledge, the presence of a C·C^+^ base pair with the structural role of a bolt has not been described for antiparallel G-quadruplex structures. Several works, on the contrary, have described similar situations for sequences containing tandem repeats of the CNG triplets (where N could be C, G, A or T). In a pioneering work it was observed that the folding of the d(CGG)_4_ sequence induced by the addition of 1 M KCl was faster at pH 5.4 than at pH 8.0^48^. The explanation of this fact was based on the initial formation of parallel G-quadruplexes aided by C·C^+^ base pair formation, which evolved to G-quadruplexes with contiguous G-tetrads and looped-out cytosines due to the high concentration of K^+^ ions. Concomitantly, Vorlickova *et al*. reported that the folding of the same sequence at pH 5 needed several hours to be completed at 25 °C and 0.07 mM DNA concentration^49^. More recently, the coexistence of C·C^+^ base pairs in small i-motif structures at neutral pH values and low temperature with tetrads resulting from the association of G:C or G:T base pairs has been reported. The interaction between the minor groove tetrads and the nearby C:C^+^ base pairs affords a strong stabilization, which results in effective pH_T_ values above 7.5^50^.

The influence of pH, sample treatment and ionic strength on the potential formation of hydrogen bonds between cytosine bases in antiparallel G-quadruplex structures formed by d[(GGGGCC)_3_GGGG] has been recently reported^51,52^. From NMR studies, it was deduced that this sequence forms two different structures (AQU and NAN) that differ in the strand orientation and pH stability. It was observed that the AQU structure is preferred over the NAN structure under slightly acidic conditions. This fact was explained as due to cytosine protonation which leads to formation of two C·C^+^ base pairs among cytosine bases present at the lateral loops that are stacked on a G-quartet. However, the presence of this base pair, whereas hypothesized, was not studied from a thermodynamic point of view and, therefore, the influence of pH and temperature on the stability of these base pairs was not fully characterized.

The C·C^+^ base pair could have a potential role *in vivo* as it provides a way to “open” or “close” G-quadruplex structures in the scenario of biological processes involving DNA structures. It should be stressed that the value of the pK_a_ (~7.1) associated with this conformational transition makes the formation of the base pair and its role as a bolt clearly accessible for the more frequent *in vivo* processes carried out at pH values around 7–7.5 and in crowding conditions. Clearly, for other situations, where pH may be even lower than 7, such as some cancer processes, the potential stabilizing role of this base pair is enhanced.

## Conclusions

In this work, the conformational equilibria of a particular guanine-rich sequence located near the promoter region of SMARCA4 gene were studied by different spectroscopic techniques and mathematical methods. It has been shown that a pair of cytosine bases located strategically at the lateral loops may act as a bolt of the structure, providing conformational homogeneity and stability that may also be further increased in simulated crowding conditions. This finding may open the door to find potential G-quadruplex-forming sequences showing cytosine bases at the loops which, in principle, would not be identified because of its potential low stability.

## Methods

### Reagents

The DNA sequences (Fig. 1c) were synthesized on an Applied Biosystems 3400 DNA synthesizer using the 200 nmol scale synthesis cycle. Standard phosphoramidites were used. Ammonia deprotection was performed overnight at 55 °C. The resulting products were purified using Glen-Pak Purification Cartridge (Glen Research). The integrity of DNA sequences was checked by means of Mass Spectrometry (Fig. S14). DNA strand concentration was determined by absorbance measurements (260 nm) at 90 °C using the extinction coefficients calculated using the nearest-neighbor method as implemented on the OligoCalc webpage^53^. Before any experiment, DNA solutions were first heated to 95 °C for 20 minutes and then allowed to reach room temperature overnight. KCl, KH_2_PO_4_, K_2_HPO_4_, HCl and LiOH were purchased from Panreac (Spain). MILLIQ water was used in all experiments. Poly(ethylene glycol) of average molecular weight 200 g·mol^−1^ (PEG200) was purchased from Sigma-Merck (Darmstadt, Germany). The absence of potential acid impurities due to PEG200 degradation that could produce acid solutions was checked previously to its use in melting experiments by measuring the pH of PEG200:water mixtures from 0 to 20% (w/v).

For NMR measurements oligonucleotides samples were prepared at a 0.15–0.40 mM concentration range, in H_2_O/D_2_O (9:1) containing 20 mM sodium phosphate buffer and 150 mM KCl, pH 6.0. The oligonucleotide samples were heated to 95 °C for 25 minute and then cooled at room temperature overnight. The pH was adjusted to 9.0 by the addition of a concentrated solution of LiOH.

### Instruments and procedures

Absorbance spectra were recorded on an Agilent 8453 diode array spectrophotometer. The temperature was controlled by means of an 89090 A Agilent Peltier device. Hellma quartz cells (1- or 10-mm path length, and 350, 1500 or 3000 µl volume) were used. Circular dichroism (CD) spectra were recorded on a Jasco J-810 spectropolarimeter equipped with a temperature control unit. Hellma quartz cells (10 mm path length, 1400 and 3000 µl volume) were used. Molar ellipticity (deg·cm^2^·mol^−1^) has been calculated according to: [Θ] = Θ/C·l, where Θ is the measured ellipticity (mdeg), C is the analytical concentration (mol·L^−1^), and l is the optical path (cm).

Spectroscopically monitored acid-base titrations were monitored by CD and/or molecular absorption spectroscopies. In all cases, experimental conditions were 20 °C and 150 mM KCl. Titrations were carried out by adjusting the pH of 1.5 mL solutions containing the oligonucleotides at 2 μM by addition of concentrated LiOH or HCl solutions. pH was measured using an Orion SA 720 pH/ISE meter and a micro-combination pH electrode (Thermo Scientific, USA). Absorbance or CD spectra were recorded simultaneously in a pH stepwise fashion by using the J-810 spectropolarimeter. Hellma quartz cells (10 mm path length, 3000 µl volume) were used.

Melting experiments were monitored either using the Agilent-8453 spectrophotometer or the Jasco J-810 spectropolarimeter, both equipped with Peltier units for temperature control. The DNA solution was transferred to a covered 10-mm-path-length cell and spectra were recorded at 2 °C intervals with a hold time of 3 minutes at each temperature, which yielded an average heating rate of approximately 0.6 °C·min^−1^. Buffer solutions were 20 mM acetate or phosphate and 150 mM KCl.

For SEC, the chromatographic system consisted of a Waters 2695 HPLC instrument equipped with a quaternary pump, a degasser, an autosampler, a photodiode-array detector with a 13-μL flow cell, and software for data acquisition and analysis. The chromatographic column used for separation at room temperature was PSS Suprema Analytical Lineal S 100–100.000 Da (PSS Polymer Standards Service GmbH, Mainz, Germany). The composition of the mobile phase was 300 mM KCl and 20 mM phosphate (pH 7.1). The flow was set to 0.8 mL·min^−1^. The injection volume was 15 μL. Blue dextran (MW 2,000,000 Da, Sigma-Merck, Darmstadt, Germany) was used as a void volume marker (5.30 mL). T_15_, T_20_, T_25_, T_20_ and T_45_ sequences were used as standards to construct the plot of logarithm of the retention time (t_R_) *vs*. molecular weight. Some standards were injected twice to assess the reproducibility of the t_R_ values, and the relative difference between t_R_ values for a given standard was lower than 0.5%. SEC profiles were normalized to equal length (Euclidean normalization) to eliminate potential variations in the DNA concentration of samples that could hinder the comparison of chromatograms. Normalization was carried out using Eq. 1 ^54^. The variable d_i_ indicates the value of absorbance at time i, whereas n is the total number of points in each chromatogram.

1 $$Normalized\,chromatogram=\frac{raw\,chromatogram}{\sqrt{{\sum }_{1}^{n}{d}_{i}^{2}}}$$

All NMR spectra were recorded on a Bruker AV600 spectrometer operating at a frequency of 600 MHz. The ^1^H spectra were acquired at a temperature ranging from 5 °C to 25 °C and were referenced to external DSS (2,2-dimethyl-2-silapentane-5-sulfonate sodium salt) set at 0.00 ppm. Chemical shifts (δ) were measured in ppm. The complete analysis could not be carried out since the presence of multiple species impedes the complete assignment of the NMR spectra.

### Data analysis

#### Melting experiments

For melting experiments, absorbance data as a function of temperature were analyzed as described elsewhere^55^. The physico-chemical model is related to the thermodynamics of DNA unfolding. Hence, for the unfolding of intramolecular structures such as those studied here, the chemical equation and the corresponding equilibrium constant may be written as:

2 $${\rm{DNA}}\,{\rm{folded}}+{\rm{heat}}\leftrightarrow {\rm{DNA}}\,{\rm{unfolded}}\,{\rm{Kunfolding}}=[{\rm{DNA}}\,{\rm{unfolded}}]/[{\rm{DNA}}\,{\rm{folded}}]$$

For melting experiments, the concentration of the folded and unfolded forms is temperature-dependent. Accordingly, the equilibrium constant depends on temperature according to the van’t Hoff equation^20^:

3 $$\mathrm{ln}\,{\rm{K}}\,{\rm{unfolding}}=-\Delta H/{\rm{RT}}+\Delta S/{\rm{R}}$$

It is assumed that ∆H and ∆S will not change throughout the range of temperatures studied here. Also, it is assumed that the transition is a two-state process, without intermediates. This assumption may be checked by means of multivariate analysis methods^56,57^.

#### Acid-base titrations

CD and molecular absorption spectra recorded along acid-base titrations were monitored in a range of wavelengths from 220 to 320 nm. Later, they were arranged in a table or data matrix **D**, with *m* rows (spectra recorded) and *n* columns (wavelengths at which ellipticity or absorption were measured). To gain insight in the definition of the acid-base equilibria and to improve the identification of the structure of the species involved, simultaneous analysis of the two data matrices **D**_**CD**_ and **D**_**abs**_ of the same sample coming from the two different techniques used was done using a row-wise augmented matrix (Fig. S12).

The goal of data analysis was the calculation of distribution diagrams and pure (individual) spectra for all *nc* components considered throughout the process. The distribution diagram provides information about the stoichiometry and stability of the acid-base components considered. In addition, the shape and intensity of the pure spectra may provide qualitative information about the structure of those components. With this goal in mind, data matrix **D** was decomposed according to Beer-Lambert-Bouer’s law in matrix form:

4 $${\bf{D}}={\bf{C}}\,{{\bf{S}}}^{{\bf{T}}}+{\bf{E}}$$

where **C** is the matrix (*m* × *nc*) containing the distribution diagram, **S**^**T**^ is the matrix (*nc* × *n*) containing the pure spectra, and **E** is the matrix of data (*m* × *n*) not explained by the proposed decomposition (Fig. S12).

The mathematical decomposition of **D** into matrices **C**, **S**^**T**^, and **E** may be conducted in two different ways, depending on whether a physico-chemical model is initially proposed (hard-modeling approach) or not (soft-modeling approach)^58^. For hard-modeling approaches, the proposed model depends on the nature of the process under study.

For acid-base experiments the model will include a set of chemical equations describing the formation of the different acid-base components from the neutral species, together with approximate values for the stability constants, such as the following:

5 $${\rm{DNA}}+{{\rm{pH}}}^{+}\leftrightarrow {\mathrm{DNA}\cdot H}_{{\rm{p}}}\,{{\rm{Beta}}}_{1{\rm{p}}}=[{\mathrm{DNA}\cdot H}_{{\rm{p}}}]/[{\rm{DNA}}]{[{{\rm{H}}}^{+}]}^{{\rm{p}}}$$

In this equation, the parameter *p* is related to the Hill coefficient and describes qualitatively the cooperativity of the equilibrium. Values of *p* greater than 1 indicate the existence of a cooperative process.

Whenever a physico-chemical model is applied, the distribution diagram in **C** complies with the proposed model. Accordingly, the proposed values for the equilibrium constants and the shape of the pure spectra in **S**^**T**^ are refined to explain satisfactorily data in **D**, whereas residuals in **E** are minimized. In this study, hard-modeling analysis of acid-base experiments used the EQUISPEC program^27^.

## Supplementary information

Supplementary Information

## Data Availability

The datasets generated during and/or analysed during the current study are available from the corresponding author on reasonable request.
