# Supplementary material for: A pH-dependent bolt involving cytosine bases located in the lateral loops of antiparallel G-quadruplex structures within the SMARCA4 gene promotor
Source: Sci Rep. 2019 Nov 1;9:15807. doi: 10.1038/s41598-019-52311-5 (PMC6825181; doi:10.1038/s41598-019-52311-5)
Supplement: Supplementary file 1 — Supplementary Information [file 41598_2019_52311_MOESM1_ESM.pdf]

Electronic Supplementary Information

A pH-dependent bolt involving cytosine bases located in the lateral loops of antiparallel G-quadruplex structures within the SMARCA4 gene promotor

Sanae Benabou[1], Stefania Mazzini [2], Anna Aviñó[3], Ramon Eritja[3], Raimundo Gargallo[1]*

Contents:

Table S1	Watson-Crick-based folding predictions based on *in silico* analysis using the mfold web server method
Table S2	G-quadruplex folding predictions based on *in silico* analysis using the QGRS mapper web server
Table S3	Influence of K^+ concentration on T_m and thermodynamic data calculated from the melting experiments of SMG03
Table S4	Influence of pH on T_m and thermodynamic data calculated from the melting experiments of SMG03 and mutants
Figure S1	Thermal Difference and CD spectra and of SMG01-SMG04 sequences
Figure S2	Acid-base titration of SMG03
Figure S3	Acid-base titration of TBA
Figure S4	Acid-base titration of SMG03T6
Figure S5	Acid-base titration of SMG03T11
Figure S6	Acid-base titration of SMG03T16
Figure S7	Heating/cooling traces of SMG03 at pH 5.0 and 6.0
Figure S8	CD and melting experiments of SMG03A7
Figure S9	Size-Exclusion chromatography
Figure S10	Imino proton region of ^{1}H NMR spectra at pH 9.0
Figure S11	Imino proton region of ^{1}H NMR spectra of SMG03 at different temperatures
Figure S12	Augmented bilinear model for a row-wise augmented matrix of the same by CD and molecular spectroscopy
Figure S13	Multivariate analysis of acid-base titrations of SMG01, SMG01T6 and SMG01T16
Figure S14	MALDI-TOF MS spectra of oligonucleotides

Table S1. Watson-Crick-based folding predictions based on *in silico* analysis using the mfold web server method. The calculations were done using the web server located in http://unafold.rna.albany.edu/?q=mfold/DNA-Folding-Form. Standard errors are roughly ±5%, ±10%, ±11% and 2-4 °C for free energy, enthalpy, entropy and T_m, respectively. Calculations were done considering 25 °C and 150 mM Na^+.

Sequence name	Proposed structure	ΔG_{25oC} (kcal·mol^{-1})	T_m(°C)
SMG01	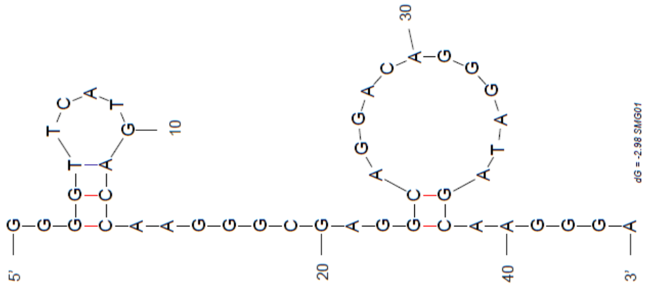	-2.98	43
	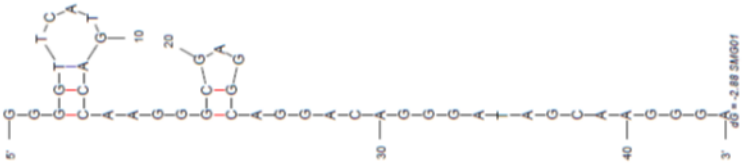	-2.88	43
	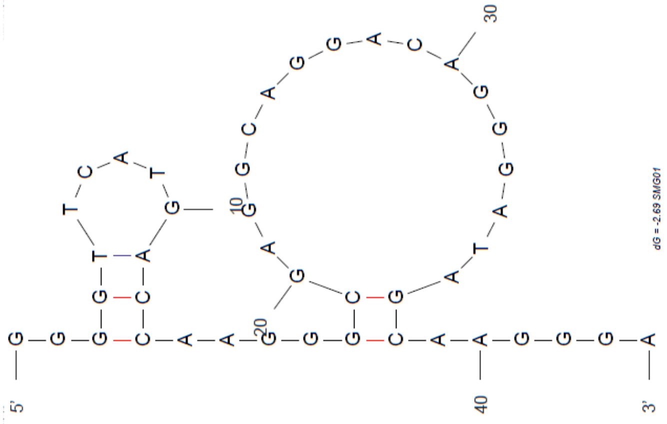	-2.69	40
SMG02	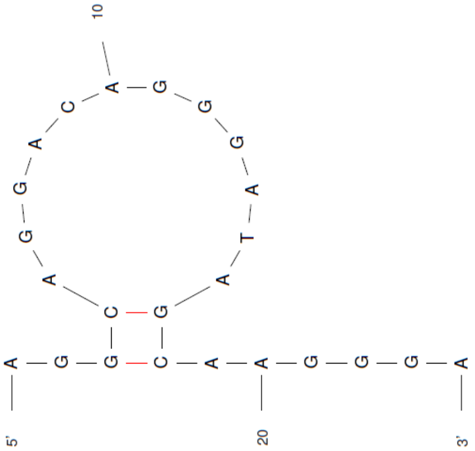	-0.56	33

SMG03	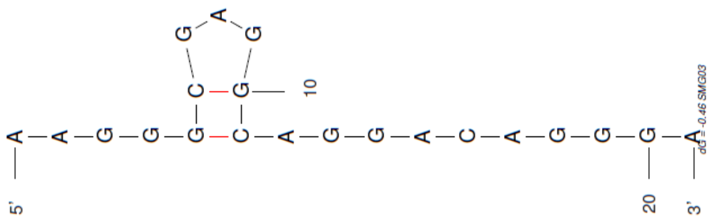	-0.46	32
SMG04	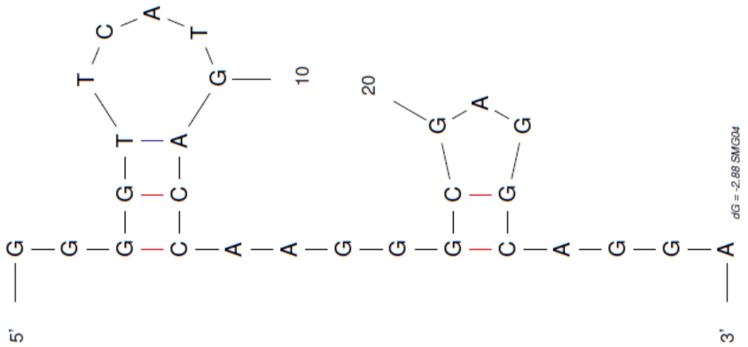	-2.88	43

Table S2. G-quadruplex folding predictions based on *in silico* analysis using the QGRS mapper web server.

The table summarizes those sequences showing the highest G-score, i.e., those that are more prone to form stable G-quadruplex structure.

The webserver is available at http://bioinformatics.ramapo.edu/QGRS/index.php. The standard search criteria have been used: QGRS maximum length (30), min G-group size (2) and loop size ranging from 0 to 36 nucleotides.

Sequence name	Position of the predicted G-quadruplex into the sequence (5'-> 3')	G-score
SMG01	GGG GTT CAT GAC CAA G**GG** CGA **GG**C A**GG** ACA **GG**G ATA GCA AGG GA	20
SMG02	A **GG**C A**GG** ACA G**GG** ATA GCA A**GG** GA	16
SMG03	AA G**GG** CGA **GG**C A**GG** ACA **GG**G A	20
SMG04	GG**G** **G**TT CAT GAC CAA **GG**G CGA **GG**C A**GG** A	12
TBA	**GG** TT **GG** TGT **GG** TT **GG**	20

The TBA sequence, which is known to form a stable G-quadruplex structure, has been included to compare the value of its corresponding G-score with those of the studied sequences.

Table S3. Influence of K^+ concentration on T_m and thermodynamic data calculated from the melting experiments of SMG03.

Thermodynamic values refer to the unfolding process. Uncertainties in T_m, ΔH, ΔS, and ΔG_{37} are ±0.7 ºC, 5%, 5% and 10%, respectively. In all cases, a two-step process has been considered. The experimental conditions were 20 mm phosphate buffer (pH 7.1) and 2 μM DNA concentration.

SMG03	T_m (ºC)	ΔH (kcal·mol^{-1})	ΔS (cal·K^{-1}·mol^{-1})	ΔG_{37} (kcal·mol^{-1})
0 mM KCl	22.8	20.3	68.5	-0.98
50 mM KCl	26.5	21.4	71.4	-0.67
100 mM KCl	32.5	22.5	73.9	-0.34
150 mM KCl	37.0	23.3	75.3	0.00

Table S4. Influence of pH on T_m and thermodynamic data calculated from the melting experiments of SMG03 and mutants

Thermodynamic values refer to the unfolding process. T_m, ΔH, ΔS and ΔG values are given in °C, kcal·mol^{-1}, cal·K^{-1}·mol^{-1}, and kcal·mol^{-1}, respectively. Uncertainties in T_m, ΔH, ΔS, and ΔG_{37} are ±0.7 °C, 5%, 5% and 10%, respectively. In all cases, a two-step process has been considered. The experimental conditions were 20 mm acetate or phosphate buffer, 150 mM KCl and 2 μM DNA concentration.

	pH 5.0				pH 6.0				pH 7.4			
	T_m	ΔH	ΔS	ΔG_{37}	T_m	ΔH	ΔS	ΔG_{37}	T_m	ΔH	ΔS	ΔG_{37}
SMG03	60.0	28.6	86	1.98	48.8	34.3	106.7	1.25	37.0	23.3	75.3	0.00
SMG03T6	53.4	31.6	96.8	1.59	38.2	34.4	110.4	0.13	38.7	28.7	92.0	0.16
SMG03T11	63.1	29.7	88.4	2.30	48.7	44.8	139.2	1.63	39.0	39.0	125.2	0.24
SMG03T16	51.4	32.4	100.0	1.44	39.2	34.9	111.7	0.25	37.0	27.8	90.1	0.00

Figure S1. Thermal Difference and CD spectra and of SMG01-SMG04 sequences

(a) Normalized TDS measured in 150 mM KCl, 20 mM phosphate buffer (pH 7.2), 2 μM DNA. (b) CD spectra measured in 150 mM KCl, 20 mM phosphate buffer (pH 7.2), 10 °C, 2 μM DNA. The arrows indicate those spectral features that have been related to the formation of an antiparallel G-quadruplex by SMG03 sequence.

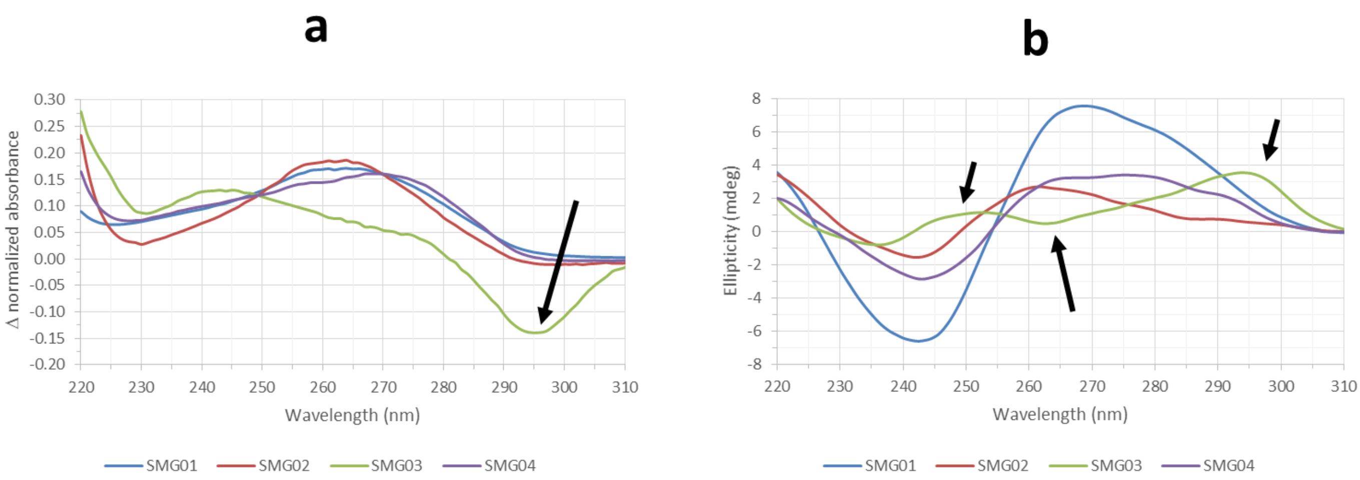

Figure S2. Acid-base titration of SMG03

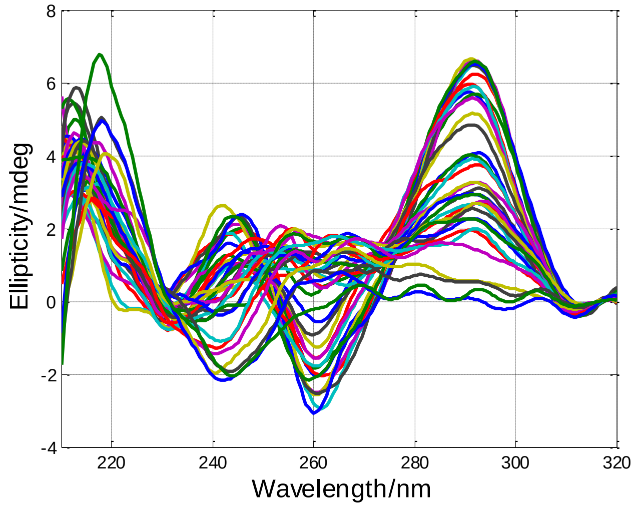

Complete set of 37 CD spectra measured from pH 2 to pH 12. Experimental conditions as detailed in Figure 2.

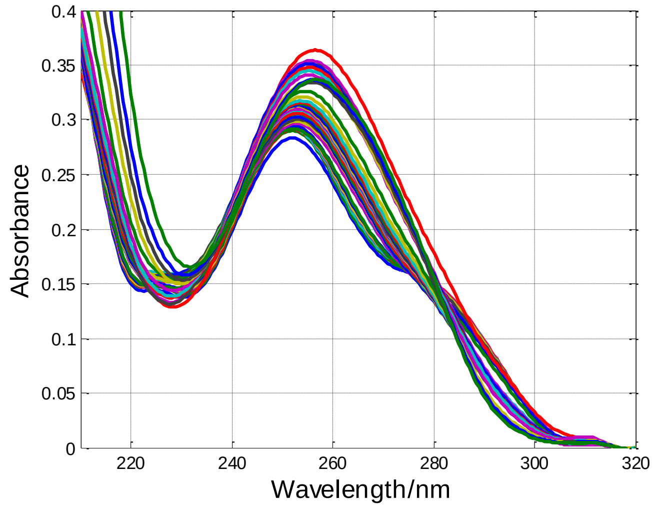

Complete set of 37 molecular absorption spectra.

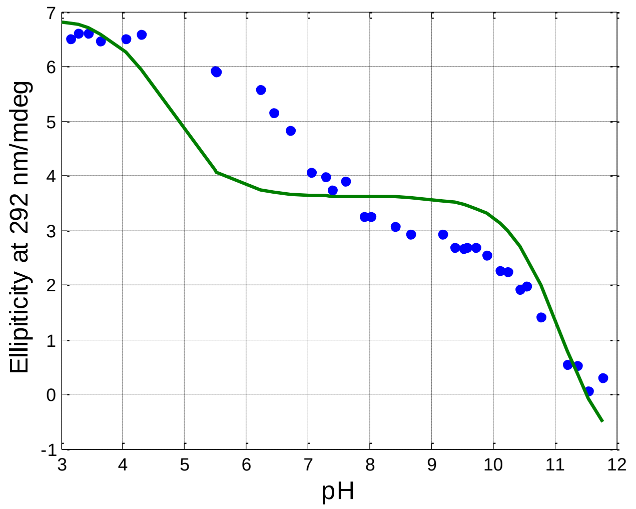

Experimental CD (blue symbols) and calculated with a proposed model of three acid-base components (green line) at 292 nm

Figure S3. Acid-base titration of TBA

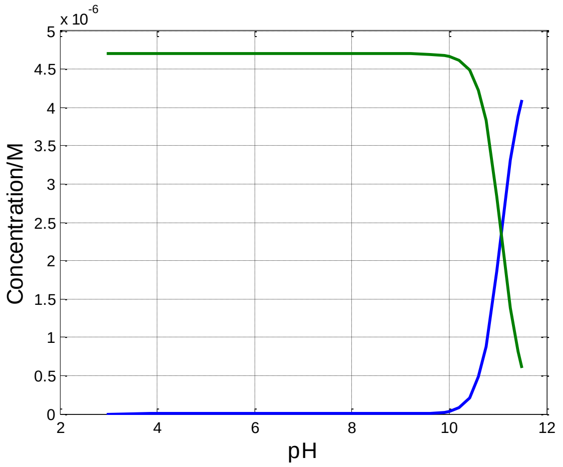

Calculated distribution diagram for the model including two acid-base components. Green: G-quadruplex showing all bases in neutral form. Blue: unfolded strand showing deprotonated guanine bases.

Experimental conditions were 150 mM KCl, 20 °C. DNA concentration was 2 μM.

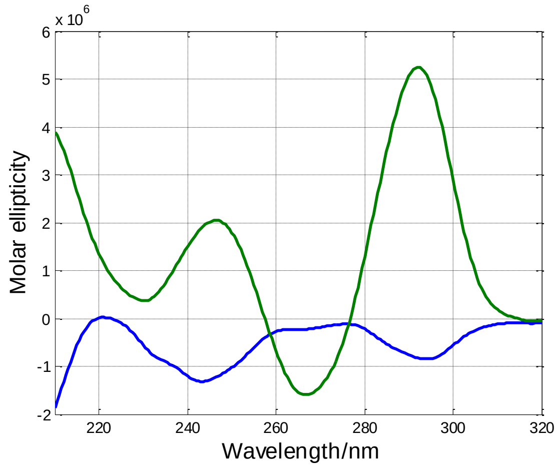

Calculated pure CD spectra for the two acid-base components. Green: TBA showing all bases in neutral form. Blue: unfolded strand showing deprotonated guanine bases.

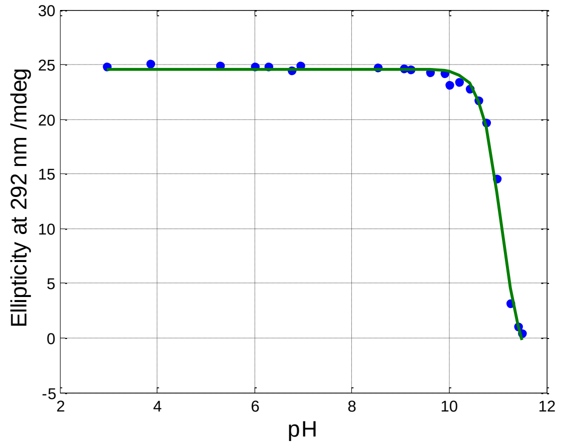

Experimental CD (blue symbols) and calculated with the proposed model of two species (green line) at 292 nm.

Figure S4. Acid-base titration of SMG03T6

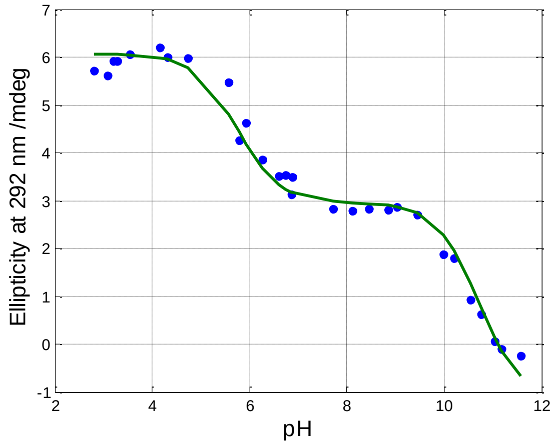

Experimental CD (blue symbols) and calculated with the proposed model of three species (green line) at 292 nm. Experimental conditions were 150 mM KCl, 20 ^{o}C. DNA concentration was 2 μM.

Figure S5. Acid-base titration of SMG03T11

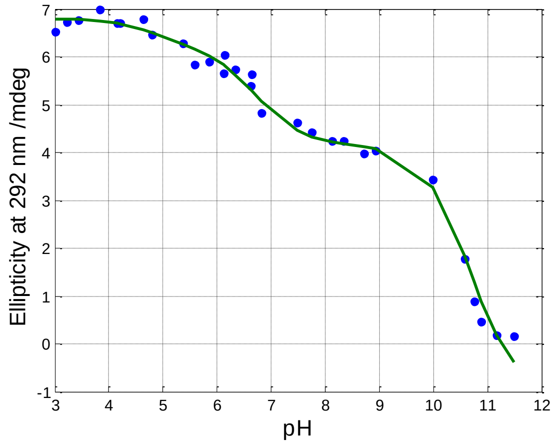

Experimental CD (blue symbols) and calculated with the proposed model of four species (green line) at 292 nm. Experimental conditions were 150 mM KCl, 20 ^{o}C. DNA concentration was 2 μM.

Figure S6. Acid-base titration of SMG03T16

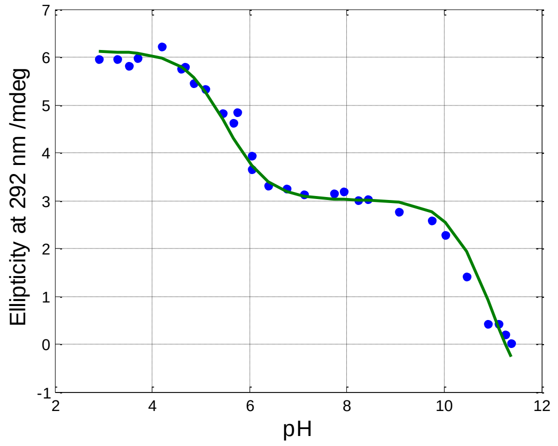

Experimental CD (blue symbols) and calculated with the proposed model of three species (green line) at 292 nm. Experimental conditions were 150 mM KCl, 20 ^{o}C. DNA concentration was 2 μM.

Figure S7. Heating/cooling traces of SMG03 at pH 5.0 and 6.0.

Experiments carried out in 20 mM acetate or phosphate buffer, 150 mM KCl, 2 μM DNA concentration. The figures present raw data without any baseline subtraction.

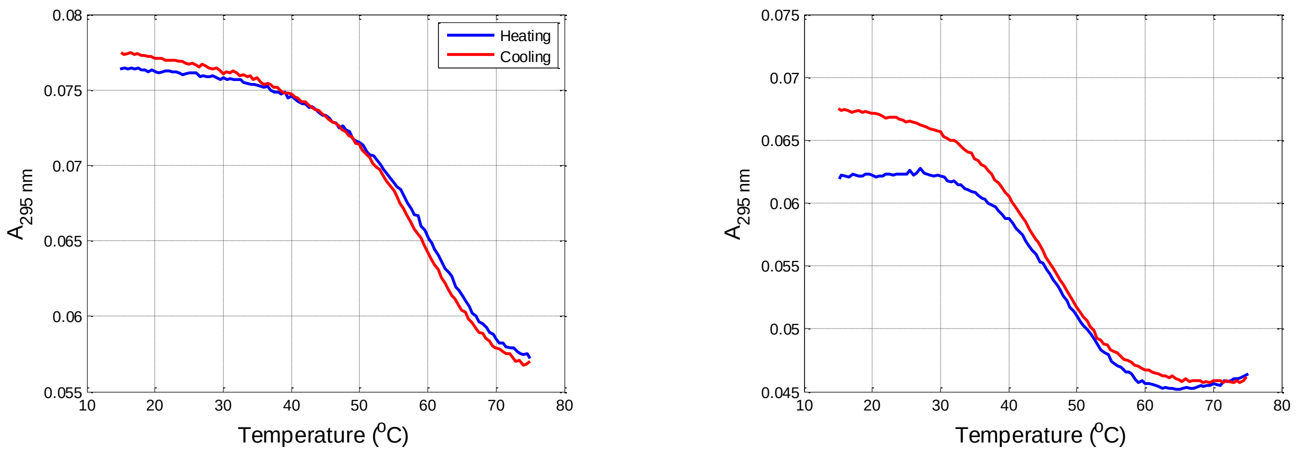

Figure S8. CD and melting experiments of SMG03A7.

CD spectra (left) and experimental and fitted melting curves using a two-state model. Experiments were carried out in 150 mM KCl, 20 mM phosphate or acetate buffer.

SMG03A7 a pH 7.4

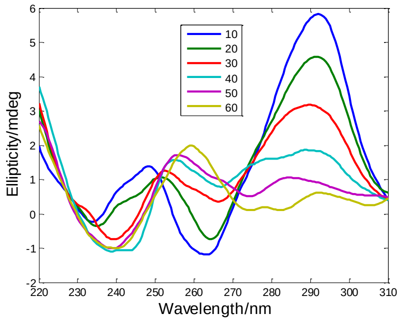

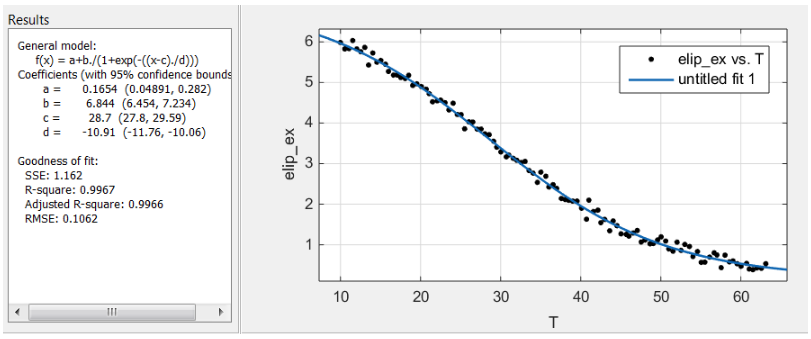

SMG03A7 a pH 4.9

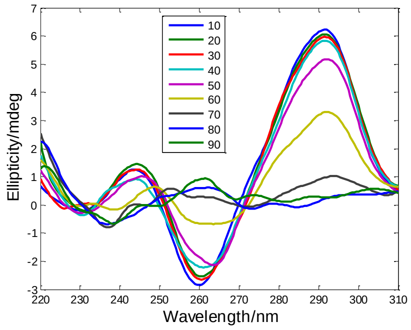

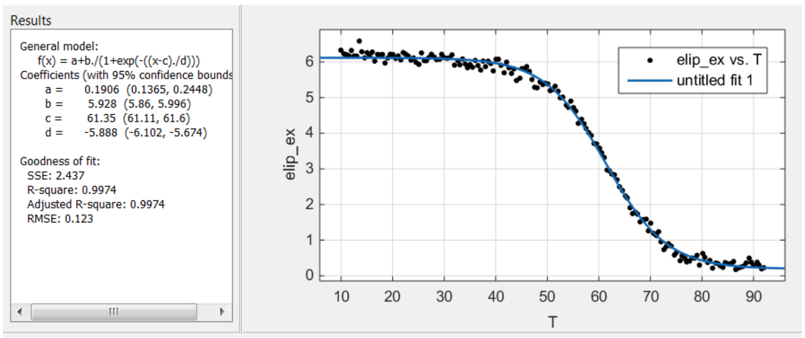

Figure S9. Size-Exclusion chromatography

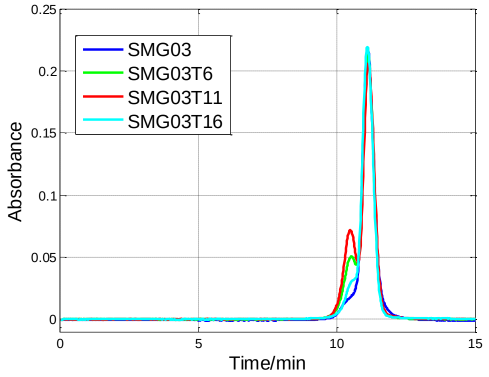

Raw chromatograms at 20 °C.

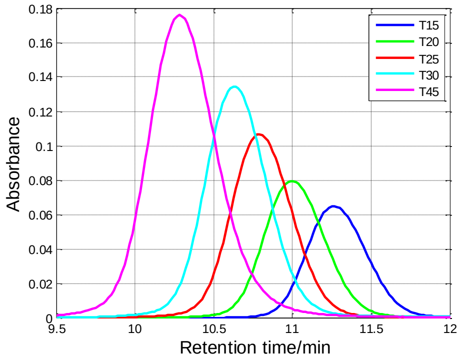

Chromatograms of Tx standards

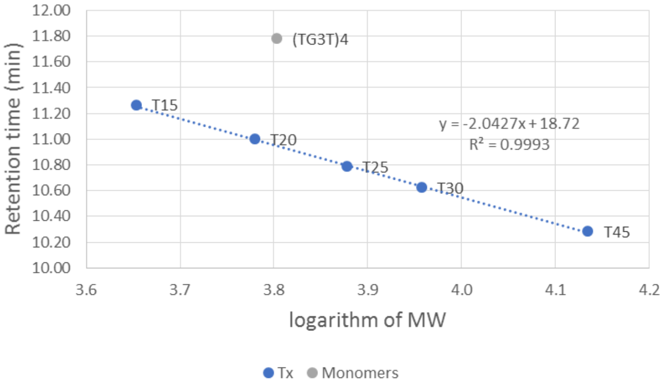

Calibration plot

Calculation of the retention time for unfolded SMG03 and mutants.

	MW	log(MW)	tR exp. pH 7	tR calc.	Variat. (%)	log(MW calc)	MW calc	ratio
SMG03	6619.3	3.82	10.50	10.92	-3.80%	4.024	10570.3	1.60
	6619.3	3.82	11.13	10.92	1.97%	3.716	5196.0	0.78
SMG03T6	6634.4	3.82	10.50	10.91	-3.79%	4.024	10570.3	1.59
	6634.4	3.82	11.13	10.91	1.99%	3.716	5196.0	0.78
SMG03T11	6634.4	3.82	10.49	10.91	-3.88%	4.029	10690.1	1.61
	6634.4	3.82	11.15	10.91	2.17%	3.706	5080.2	0.77
SMG03T16	6634.4	3.82	10.53	10.91	-3.51%	4.009	10218.8	1.54
	6634.4	3.82	11.10	10.91	1.71%	3.730	5374.7	0.81

Figure S10. Imino proton region of ^{1}H NMR spectra at pH 9.0.

(a) SMG03, (b) SMG03T11 and (c) SMG03T6 at 25°C, 20 mM phosphate buffer and 150 mM KCl, 0.15 mM DNA concentration.

(c)

(b)

(a)

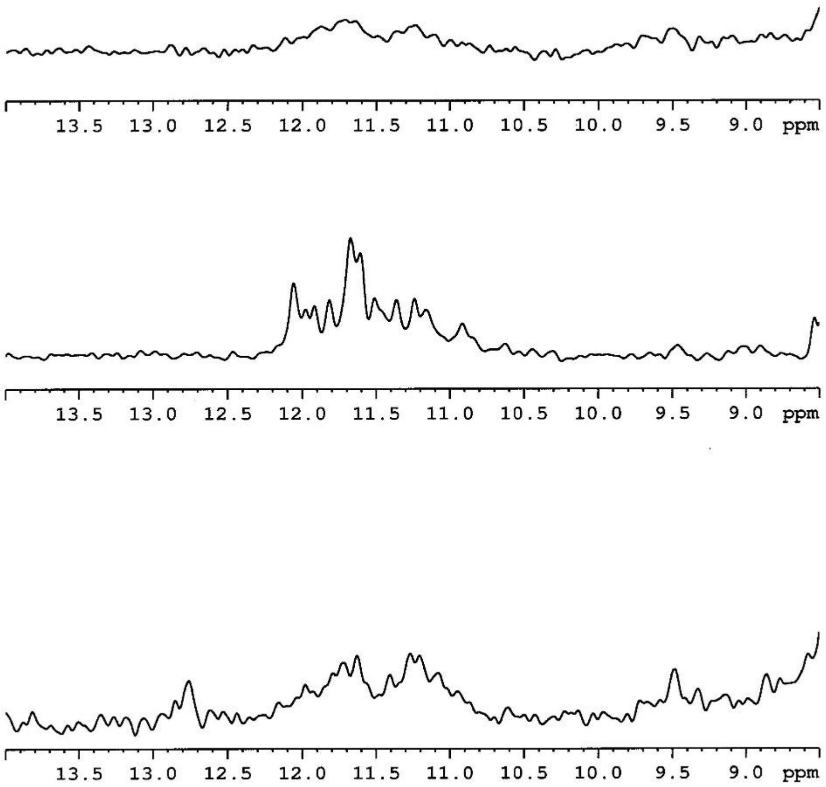

Figure S11. Imino proton region of ^{1}H NMR spectra of SMG03 at different temperatures.

(a) 25°C, (b) 10°C and (c) 5°C, 20 mM phosphate buffer and 150 mM KCl, pH 6.0, 0.15 mM DNA concentration.

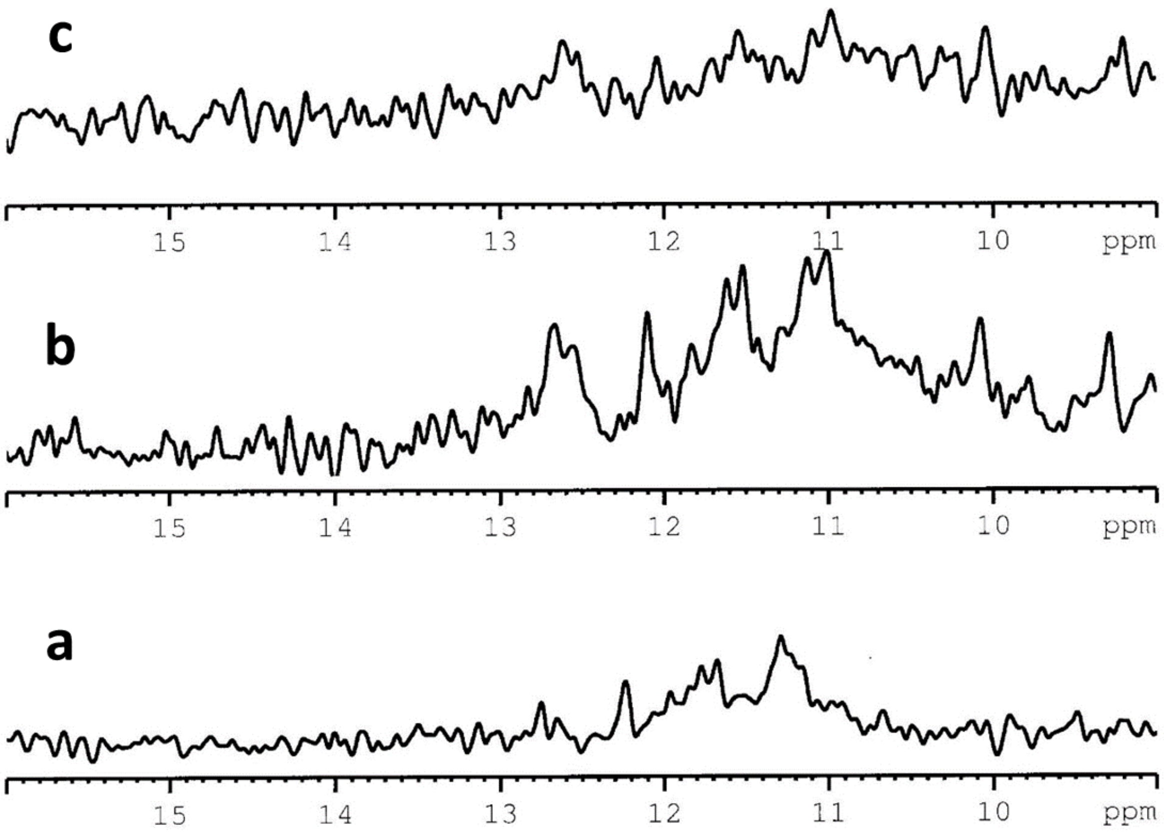

Figure S12. Augmented bilinear model for a row-wise augmented matrix of the same by CD and molecular spectroscopy.

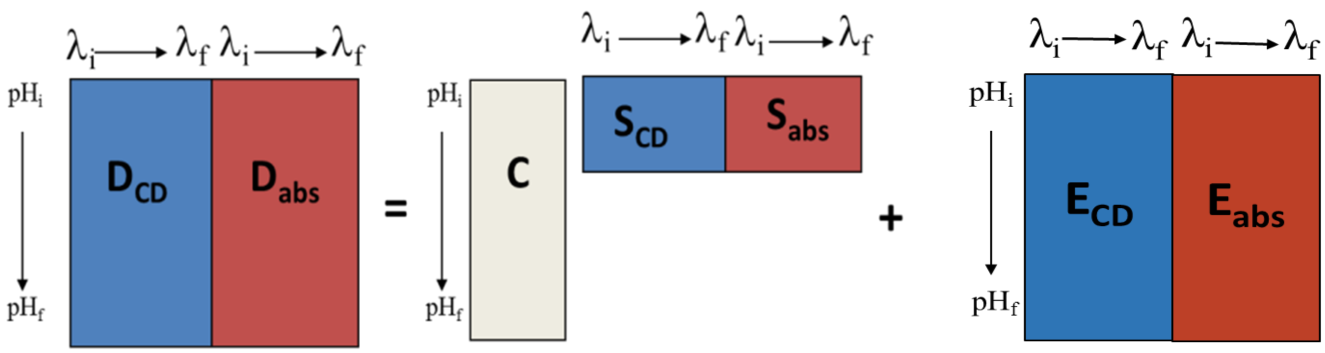

Figure S13. Multivariate analysis of acid-base titrations of SMG01, SMG01T6 and SMG01T16.

Calculated distribution diagrams (a, c, and e) and CD spectra (b, d, and f) for each one of the acid-base components considered in the multivariate analysis of spectra measured along the titrations of SMG01 (a, b), SMG01T6 (c, d) and SMG01T16 (e, f).

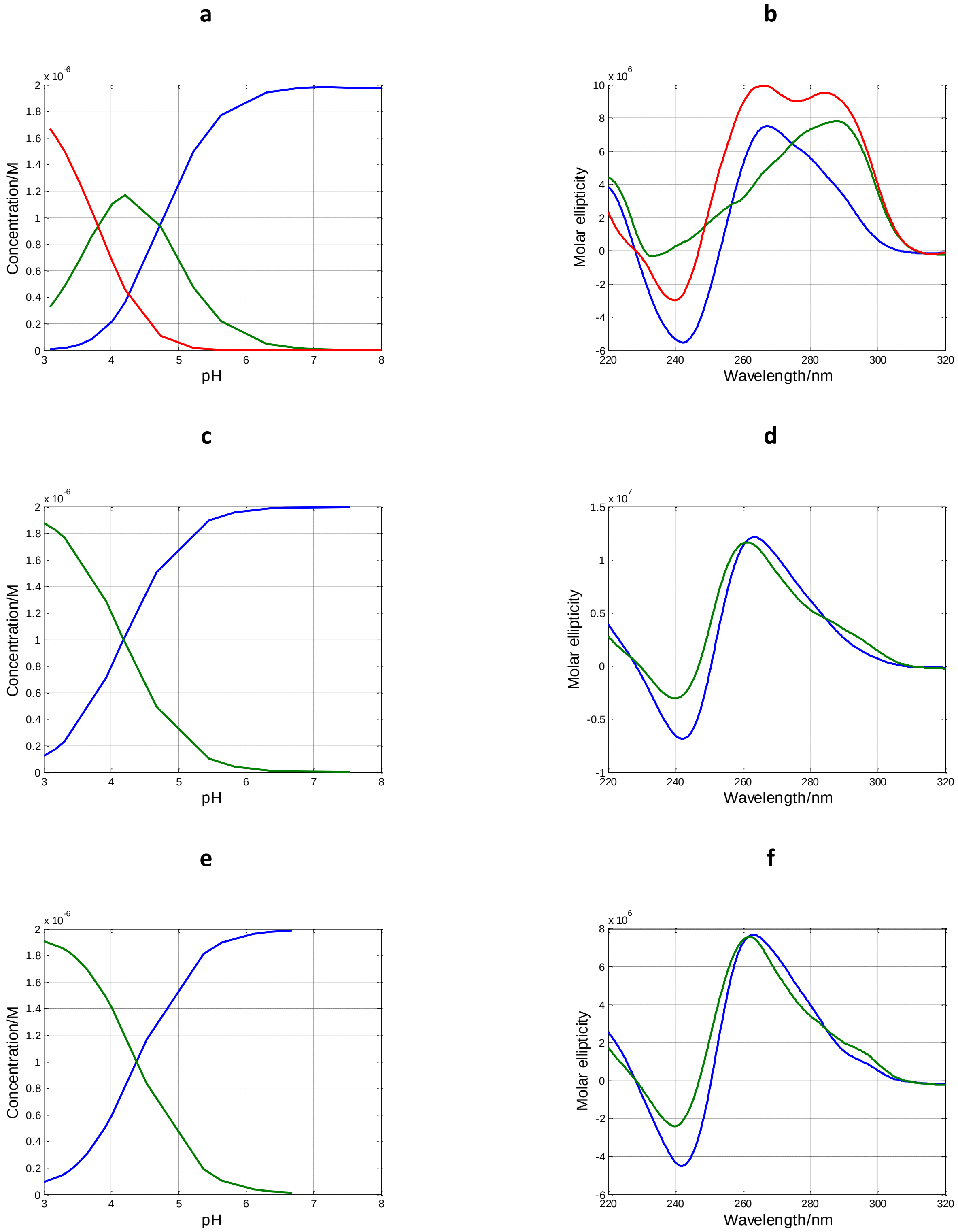

Figure S14. MALDI-TOF MS spectra of oligonucleotides.

To analyse Oligonucleotides mass spectra were recorded on a Matrix-assisted laser desorption ionization (MALDI) Voyager DETM RP time of-flight (TOF) spectrometer (Applied Biosystems, USA).

Sequence	$MW_{calculated}$	$MW_{determined}$
SMG01	13835.0	13836.3
SMG02	7750.0	7756.1
SMG03	6619.3	6626.0
SMG04	8768.7	8774.5
SMG03T6	6634.4	6634.9
SMG03T11	6634.4	6634.0
SMG03T16	6634.4	6636.0
SMG03A7	6603.3	6600.3

SMG01

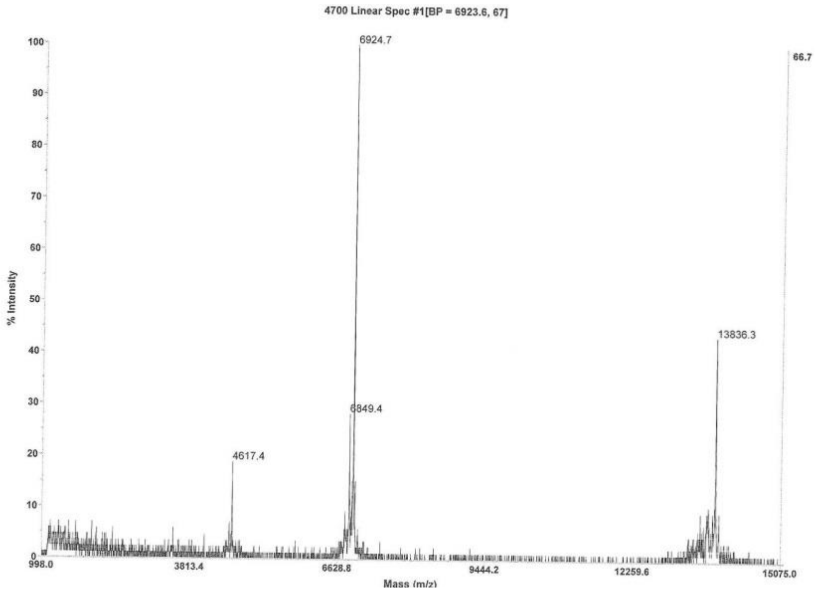

SMG02

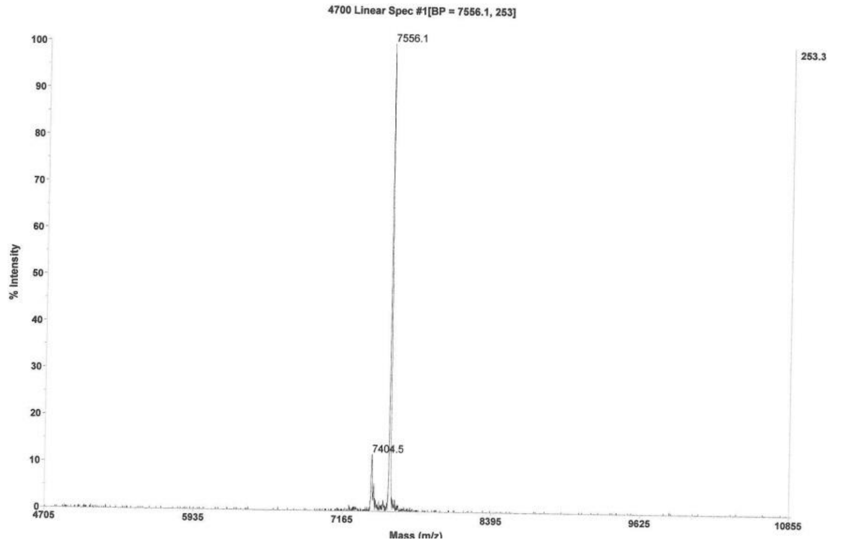

SMG03

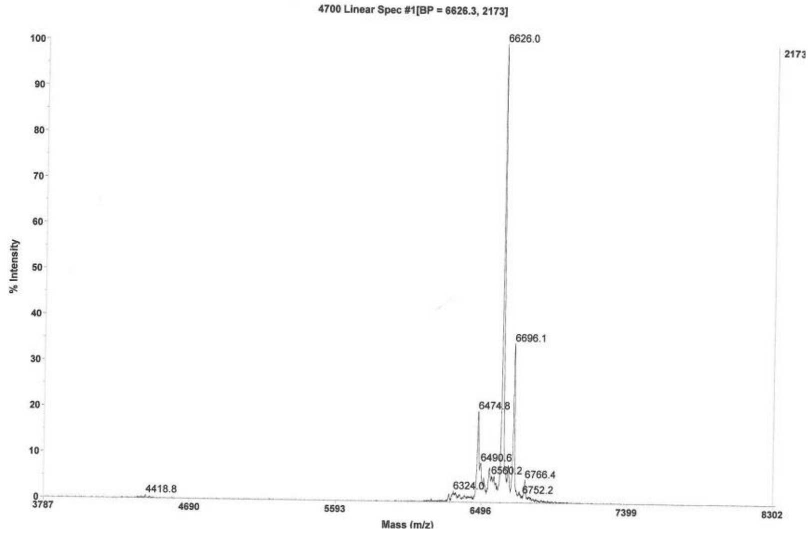

SMG04

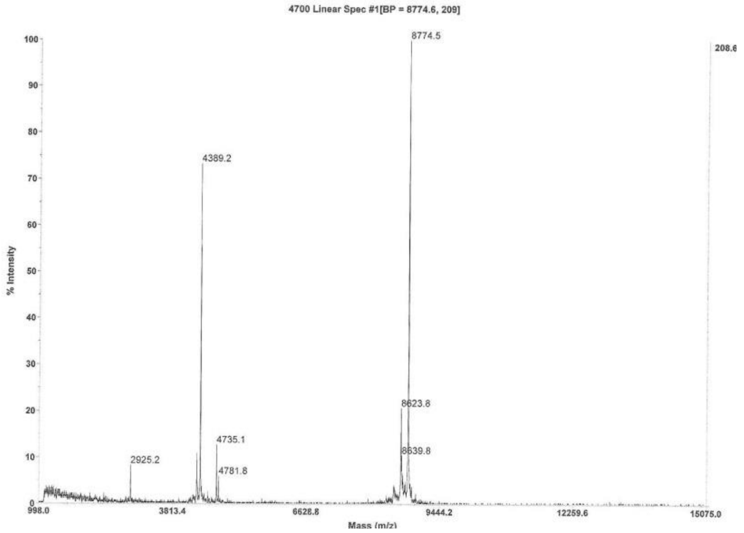

SMG03T6

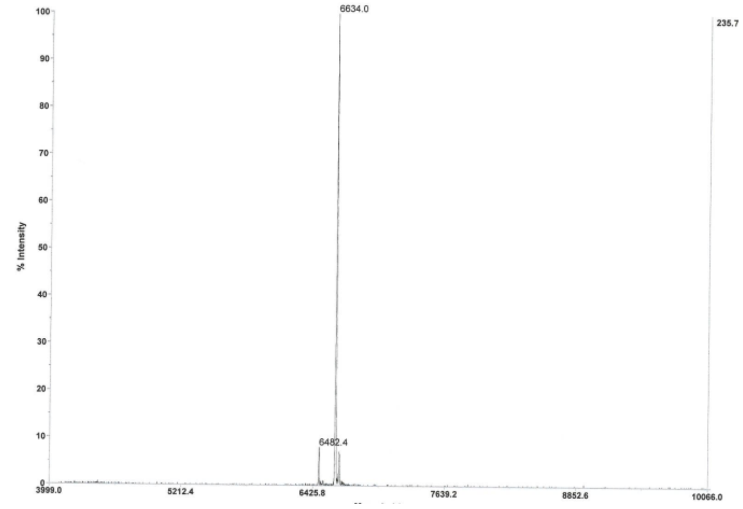

SMG03T11

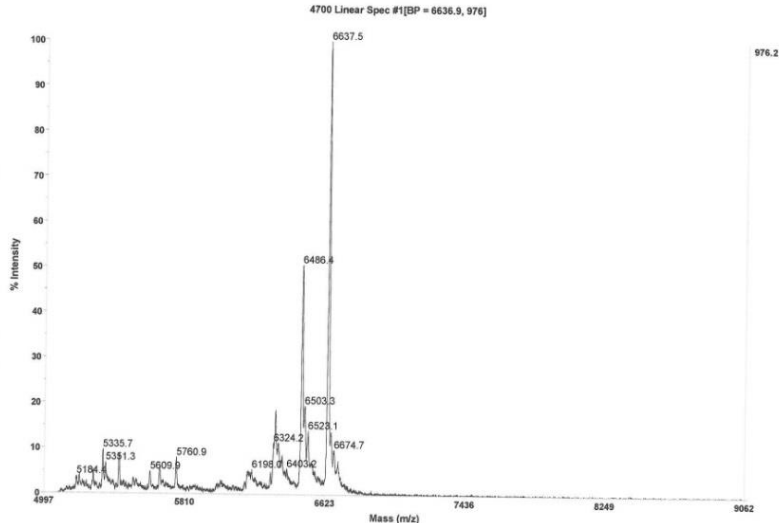

SMG03T16

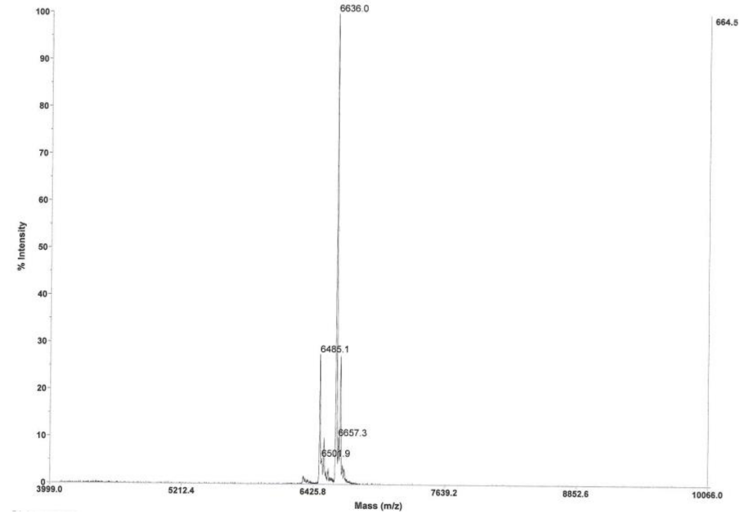

SMG03A7

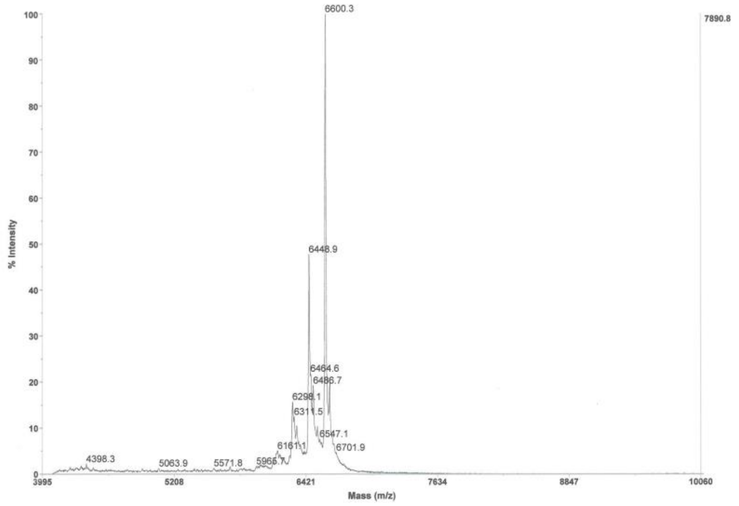

6600.3
7890.8
6448.9
6464.6
6486.7
6298.1
6311.5
6547.1
6161.1
6701.9
4398.3
5063.9
5571.8
% Intensity
Mass (m/z)
3995
5208
6421
7634
8847
10060
